# Persistent Neuronal Activity in Anterior Cingulate Cortex Correlates with Sustained Attention in Rats Regardless of Sensory Modality

**DOI:** 10.1038/srep43101

**Published:** 2017-02-23

**Authors:** Dingcheng Wu, Hanfei Deng, Xiong Xiao, Yanfang Zuo, Jingjing Sun, Zuoren Wang

**Affiliations:** 1 Institute of Neuroscience, CAS Center for Excellence in Brain Science, State Key Laboratory of Neuroscience, Shanghai Institutes for Biological Sciences, Chinese Academy of Sciences, Shanghai 200031, China; 2 Graduate School of University of Chinese Academy of Sciences, Beijing 100049, China

## Abstract

The anterior cingulate cortex (ACC) has long been thought to regulate conflict between an object of attention and distractors during goal-directed sustained attention. However, it is unclear whether ACC serves to sustained attention itself. Here, we developed a task in which the time course of sustained attention could be controlled in rats. Then, using pharmacological lesion experiments, we employed it to assess function of ACC in sustained attention. We then recorded neuronal activity in ACC using multichannel extracellular recording techniques and identified specific ACC neurons persistently activated during the period of attention. Further experiments showed that target modality had minimal influence on the neuronal activity, and distracting external sensory input during the attention period did not perturb persistent neuronal activity. Additionally, minimal trial-to-trial variability in neuronal activity observed during sustained attention supports a role for ACC neurons in that behavior. Therefore, we conclude that the ACC neuronal activity correlates with sustained attention.

The ability to maintain attention is fundamental to daily life, allowing human beings to concentrate cognitive faculties on critical tasks over prolonged periods of time^1^,^2^. Given that our environment is often complex, the brain chooses what to process over a period of time until a task is complete. Deficits in sustained attention, however, affect a large number of people, especially children with attention deficit hyperactivity disorder (ADHD), leading to difficulties in learning and in social and affective functions. Therefore, it is critical to identify neuronal mechanisms underlying sustained attention.

Many lines of evidence from studies of humans^3^,^4^,^5^,^6^,^7^,^8^, other primates^9^,^10^, and rodents^11^,^12^,^13^ indirectly support the idea that the anterior cingulate cortex (ACC) functions in sustained attention. Those reports indicate that the ACC is recruited to regulate conflict between an object of attention and distractors during goal-directed sustained attention^3^,^4^,^5^,^7^,^10^. However, some argue that attention and conflict regulation are processed separately^14^, while others propose that the ACC encodes both preparatory attention and error detection^11^,^12^,^13^, and also functions in predicting upcoming events^7^,^9^.

Using a three-choice serial reaction time task in rats, Totah and colleagues demonstrated that a subset of ACC neurons was recruited in preparatory attention^11^. In addition, Weissman and colleagues found that reduced ACC activity accounted for attention lapses^15^. Furthermore, analysis of event-related potentials (ERPs) suggests that the contingent negative variation (CNV) is caused by sustained attention^16^,^17^ and derived primarily from ACC activity^8^. Nonetheless, it remains unknown whether ACC neurons are required for maintenance of attention. Given that CNV activity persists in sustained attention, it is reasonable to predict that at least a group of ACC neurons are consistently activated or suppressed during attention.

Sustained attention is described as a psychological state of readiness to detect upcoming rare or unpredictable signals^1^, suggesting that attention enhances signal detection accuracy. Target unpredictability requires that attention be maintained^1^, while predictability of type of target enhances accuracy in tasks of attention^18^,^19^. Thus, changes in target modality may perturb attention, requiring comparisons across modalities to identify a component common to sustained attention.

To conduct such comparisons, we evaluated behavioral performance during sustained visual and olfactory attention. Using pharmacological lesion and electrophysiological experiments, we found that rat ACC neurons function in sustained attention regardless of target modality, For this analysis, we developed a training system to control the time course of sustained attention in visual and olfactory modalities, allowing evaluation of neuronal function in that period. *In vivo* multichannel recording in behaving rats revealed that the time window of activity of particular ACC neurons coincided with that of sustained attention regardless of target modality, suggesting that these neurons are critical to maintain sustained attention.

## Results

### Acquisition of sustained attention in visual and olfactory modalities in rats

**Figure 1:**
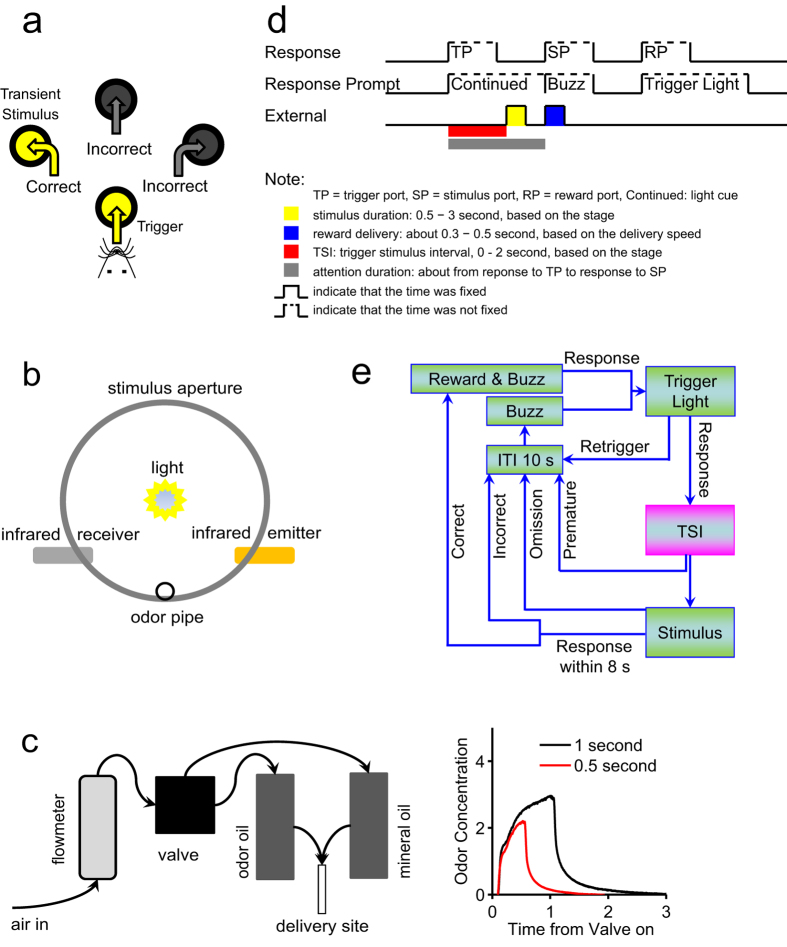
Stimulus delivery apparatus and behavioral test design. (**a**) Schematic diagram of the task panel. The water tray was located on the opposite site of the four apertures. (**b**) Schematic of stimulus aperture. Aperture depth was 2.5 cm, and the light was located at the center of the aperture base. The infrared emitter and receiver were located 0.6 cm from the entrance. For olfactory tasks, the odorant (isoamyl acetate, IAA for short) was delivered at the bottom of the entrance. (**c**) Setup for odorant delivery. Left: schematic showing components used for odorant delivery. Right: Odorant concentration at the odor outlet. When the valve was open, air passed through mineral oil containing dissolved IAA, allowing its delivery. When the valve was closed, air passed through mineral oil alone. The air flow rate was about 0.8 liter per minute. (**d**) Schematic representation of trial events. (**e**) Illustration of the procedure. As training stages advanced, TSIs (0–2 seconds) became longer and the stimulus delivery period (3–0.5 seconds) became shorter. For the test task, TSIs of 1, 1.5 or 2 seconds were used at random. Four responses were possible: correct, animal poked stimulus aperture; incorrect, animal poked non-stimulus aperture; omission, animal did not poke any aperture; and premature, animal poked stimulus aperture before stimulus delivery. “Retrigger” indicated that the trial would be stopped if animal poked the trigger twice within the TSI. Reward-associated buzz was used to speed initiation of a new trial when the rat did not respond correctly.

We designed a task to assess sustained attention in visual and olfactory modalities in rats (Fig. 1). In it, animals poked an aperture with their nose to trigger transient delivery of a stimulus from one of three other apertures after a random time interval termed the Trigger-Stimulus Interval (TSI, time window from entering the trigger port to stimulus presentation). Specifically, rats with limited access to water were first trained to detect location of the transient stimulus and then respond by poking the stimulus aperture to obtain a water reward. By increasing the TSI and decreasing stimulus duration (Fig. 1d,e, Figure S1a), tasks became more difficult but a state of attention was initiated^20^. Rats that passed the highest stage of the training procedure (Figure S1b,c) were subjected to test sessions.

Several criteria must be fulfilled to assure that a task sufficiently initiates a state of attention and its design assesses how well that state sustained. First, TSIs should differ and be randomly employed in each session. To meet this criterion, we designed three TSIs (short, medium or long) with defined gaps. Second, accuracy, defined as percentage of correct responses relative to total number of correct and incorrect trials, should not be less than 80%, and the percentage of premature trials, trials with repeated triggers (retriggers) or omitted trials should be less than 20%. We compared two types of experimental design with different TSI gaps (Figure S2d,e) and found that accuracy significantly decreased when TSIs were 3 seconds in both visual and olfactory modalities (Figure S2e, TSIs: 0, 1.5, and 3 seconds), suggesting that TSIs should be shorter than 3 seconds. Using TSIs of 1, 1.5, and 2 seconds, we observed no significant differences among the three TSIs (Figure S2d), including results relevant to premature responses, omission, and retriggers (Figure S2a). We conclude that these TSIs set met the second criterion. Third, use of different TSIs should alter the duration of attention, as defined as the time of the TSI plus response time (the duration from stimulus presentation to response). Use of short, medium and long TSIs resulted in significant differences: the longer the TSI, the more rapidly rats responded (Figure S2b), suggesting that gaps in TSI length should be great enough to assess longer attention durations associated with longer TSIs. As shown in Figure S2c, comparisons of attention duration indicated that TSI lengths employed in the designed task met these criteria, namely, TSI gaps were sufficient to distinguish three attention durations without loss of accuracy.

We also tested memory of the task. Time intervals between tests were at least six weeks (47.4 ± 6.5 days), and each test consisted of two 30-minutes sessions on two consecutive days. The results showed that rats maintained a memory of the task for at least six weeks (Figure S2f).

Overall, we conclude that the task employed is valid to assess sustained visual and olfactory attention in rats and evaluate neuronal mechanisms underlying attention.

### ACC lesions impair sustained attention regardless of modality

**Figure 2:**
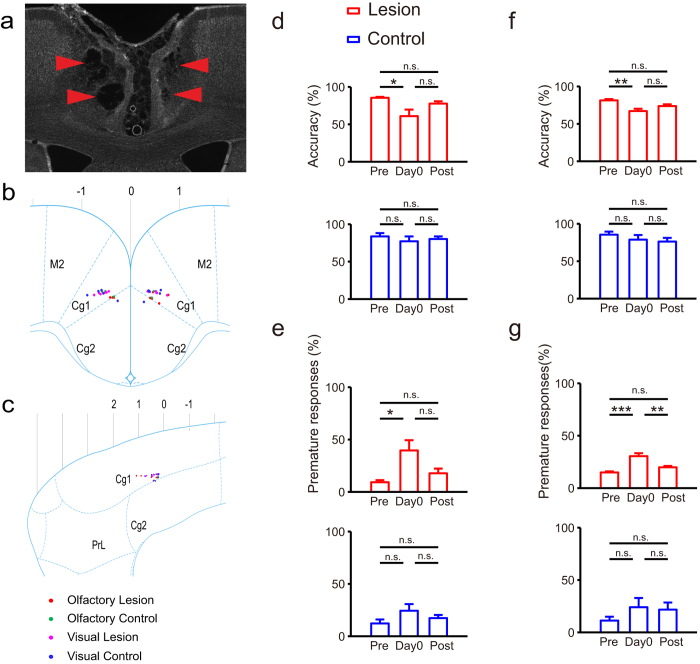
Effect of ACC lesions on sustained attention in visual and olfactory modalities. (**a**) An example of ACC with lesion by injection of Ibotenic acid (IBO). (**b,c**) IBO or control saline injection sites. Sites depicted here were deduced from coordinates relative to bregma (coordinate: 0, 0, 0) recorded in the surgery procedure and confirmed by histological methods (see panel a). Rat brain sketches were from Paxinos and Watson, 2007. (**b**) Injection sites targeting sagittal sections of rat brain (lateral 0.18 mm). (**c**) Injection sites targeting coronal sections of rat brain (bregma 0.48 mm). (**d–g**) Comparisons in accuracy or proportion of premature responses among different time. Top: comparisons in lesion group. Bottom: comparisons in control group. Pre: averaged behavioral performance on five test days before surgery; Day 0: behavioral performance on first post-surgery day; Post: averaged behavioral performance on five test days after first post-surgery day. (**d**,**e**) Visual attention (Total: n = 11, lesion: n = 6, control: n = 5). (**d**) Lesion effects in accuracy (one-way ANOVA: Lesion: *F*(2,15) = 5.41, *P* = 0.017, Control: *F*(2,12) = 0.44, *P* = 0.657). (**e**) Lesion effects in premature responses (one-way ANOVA: Lesion: *F*(2,15) = 6.16, *P* = 0.011, Control: *F*(2,12) = 1.78, *P* = 0.210). (**f**,**g**) Olfactory attention (Total: n = 9, lesion: n = 5, control: n = 4). (**f**) Lesion effects in accuracy (one-way ANOVA: Lesion: *F*(2,12) = 8.56, *P* = 0.005, Control: *F*(2,9) = 0.87, *P* = 0.452). (**g**) Lesion effects in premature responses (one-way ANOVA: Lesion: *F*(2,12) = 17.82, *P* = 0.0003, Control: *F*(2,9) = 1.00, *P* = 0.407). The results of multiple comparisons showed in panel d-g were from post hoc of one-way ANOVA with Bonferroni adjustment.

Previous rat studies employing the 5-choice serial reaction time test (5CSRTT) show that ACC lesions promote long-term loss of preparatory visual attention^12^,^13^. Lack of lesion data relevant to other modalities led us to assess ACC function in both visual and olfactory attention following ACC lesioning by ibotenic acid injection (Fig. 2a–c). We began test sessions approximately 6 (5.8 ± 1.7) days after lesion surgery. We first compared accuracy and proportion of premature responses (based on one-way ANOVA) in tests undertaken (1) pre-surgery (Pre), (2) on the first post-surgery day (day 0), and (3) after day 0 (Post). Overall, ACC lesioning significantly decreased accuracy in both visual (Fig. 2d) and olfactory (Fig. 2f) attention, and significantly increased the probability of premature responses in both visual (Fig. 2e) and olfactory (Fig. 2g) attention tasks. However, we observed no significant differences between tests of Pre and Post in accuracy or proportion of premature responses regardless of modality (Fig. 2d–g). Thus, sustained attention deficits seen following ACC lesioning recovered over time in post-surgery tests (Figure S3c,d). Saline-injected controls showed no significant effects relative to the lesion group (Fig. 2d–g).

Direct comparisons of lesion and control groups revealed significant differences in accuracy on day 0 in all rats in both visual and olfactory tasks (Figure S3a). Relevant to the proportion of premature responses, we observed no significant differences in tests on day 0 (Figure S3b).

### ACC neuronal activity correlates with sustainment of attention regardless of modality

**Figure 3:**
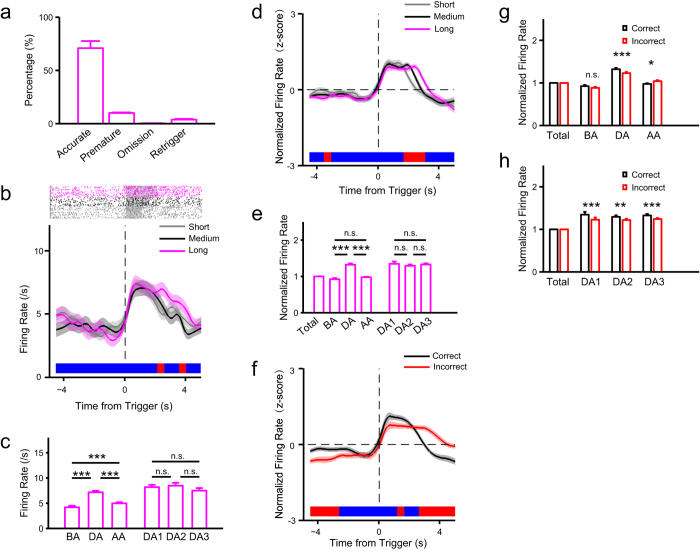
ACC neuronal activity correlates with visual sustained attention. (**a**) Behavioral performance in the recording sessions (rat no. = 4). (**b**) Activities of an attention-related activated neuron (#157). Top: raster plot of spikes at the three TSI values; grey transparent shadow indicates TSI time window; bottom: trend of firing rate in correct trials at the three TSI values aligned to the time from trigger. (**c**) Comparisons in firing rate of neuron #157 among time windows: duration of attention (DA), an interval equaling DA before attention (BA), and the same interval after attention (AA) (one-way ANOVA: *F*(2,360) = 85.04, *P* = 5.66e-31). The DA period was divided into three equal and consecutive time windows (DA1, DA2 and DA3) (one-way ANOVA: *F*(2,360) = 0.78, *P* = 0.460). (**d**) Population activities of attention-related activated neurons shown by trend of normalized firing rate in correct trials at the three TSI values aligned to the time from trigger (n = 46, proportion: 13.11%, all recorded neurons: n = 351). (**e**) Comparisons in normalized firing rate of all attention-related activated neurons among time windows (see **c**, one-way ANOVA: BA, DA, and AA: *F*(2,135) = 65.41, *P* = 1.37e-20). For each time window, the firing rate was normalized with mean firing rate of the total time window (from −5 to 5 second aligned to trigger). (**f–h**) Comparisons between correct and incorrect trials for all attention-related activated neurons (same as **d**). (**f**) Trend of normalized firing rate aligned to the time from trigger. (**g**) Comparisons in normalized firing rate (see **e**) in the three time windows BA, DA and AA (see **c**) between correct and incorrect trials. (**h**) Comparisons in normalized firing rate (see **e**) in the three time windows DA1, DA2 and DA3 (see **c**) between correct and incorrect trials. The results of multiple comparisons showed in panel c and e were from post hoc of one-way ANOVA with Bonferroni adjustment. The statistic results showed in panel g and h were from post hoc simple effect analyses (MANOVA).

To further assess ACC function in sustained attention, we recorded neuronal activity using multichannel extracellular recording techniques (Figure S4) while rats performed a visual task. After sorting isolated neurons using MClust software (Figure S5), we searched for neurons showing altered activity during sustained attention, beginning when the animal poked the trigger port. Neurons activated or suppressed during that period were identified based on whether their activity during time window of attention significantly changed relative to the time window before attention (paired *t*-test, *P* < 0.05, e.g., Fig. 3b,c). The activated population contained three types of neurons based on activity at the trigger (Figure S7). Evaluation of their average activity indicated persistent activation during attention because of no significant differences in early, middle or late periods of attention duration (Fig. 3e, one-way ANOVA: *F*(2,135) = 0.31, *P* = 0.732). It is noteworthy neuronal activity after the response in the short TSI condition differed among the three TSI conditions (Fig. 3d), confirming that activity persists during a period of sustained attention. Activity of the suppressed population persisted over the attention period (Figure S8d), suggesting that these neurons also function in sustained attention. However, neurons suppressed during attention became activated when rats began consuming the reward (Figure S8f), suggesting that their activity is also correlated with reward.

We then compared correct and incorrect trials, as previous studies indicate that neuronal activity in incorrect trials could be decreased relative to correct trials during attention processing^11^,^21^,^22^. In terms of activated neurons, neuronal activity in incorrect trials was sustained longer than in correct trials (Fig. 3f, Figure S9a–c). Simple effect analyses after two-way ANOVA with Greenhouse-Geisser adjustment (correctness: *F*(1,45) = 2.83, *P* = 0.099; time: *F*(2,90) = 59.37, *P* = 2.42e-14; correctness × time: *F*(2,90) = 12.22, *P* = 1.31e-4) showed that during the attention period neuronal activity was significantly higher for correct trials relative to incorrect trials, and in the time period after the attention task neuronal activity was significantly higher for incorrect trials relative to correct trials (Fig. 3g,). Further, significant differences between correct and incorrect trials showed in early, middle or late periods of attention duration (Fig. 3h, two-way ANOVA with Greenhouse-Geisser adjustment: correctness: *F*(1,45) = 29.45, *P* = 2.20e-6; time: *F*(2,90) = 0.25, *P* = 0.678; correctness × time: *F*(2,90) = 0.78, *P* = 0.429). Comparisons of premature and correct trials showed similar results (Figure S10a–c). Evaluation of suppressed neurons also revealed sustained activity after the response in incorrect trials but not in correct trials, although no significant differences were seen during the sustained attention (Figure S13a–c). These results may be due to the fact that in incorrect and premature trials an attention state was maintained since no reward was delivered.

We then compared visual and olfactory sustained attention and corresponding neuronal activity and recorded neuronal activity while rats performed an olfactory attention task. We then searched for neurons activated or suppressed during sustained attention and performed population analyses on these neurons. Results seen following olfactory attention were similar to those following visual attention: (1) persistent neuronal activity during sustained olfactory attention was seen in both activated (Figure S11d,e, Figure S12a–c) and suppressed (Figure S12d–f) neurons; (2) activity of these neurons in incorrect and premature trials was also prolonged after the response (Figure S9d–f, Figure S10d–f).

**Figure 4:**
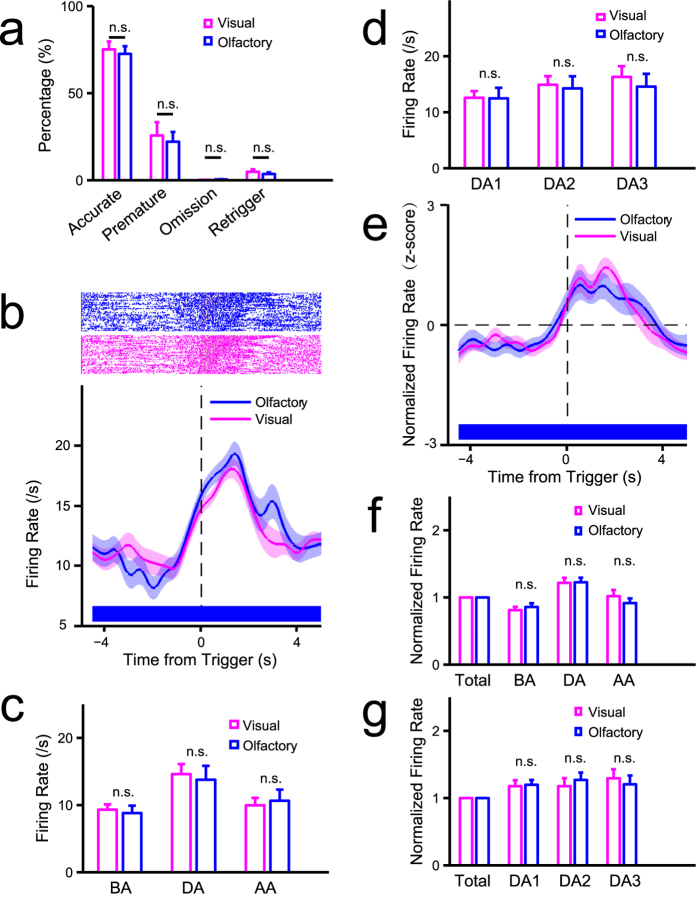
The neuronal basis of sustained attention in ACC is comparable in different sensory modalities. (**a**) Comparisons in behavioral performance between visual and olfactory attention (n = 11, 5 rats first trained in olfactory task, 6 first in visual task). (**b–g**) Electrophysiological data (rat no. = 2). (**b**) Comparisons in activity of an attention-related activated neuron (#5) between different modalities. Top: raster plots of spikes, grey transparent shadow indicates TSI time window; bottom: trend of firing rate in correct trials aligned to the time of trigger onset. (**c**) Comparisons in firing rate of neuron #5 in the time windows of DA, BA, and AA (see Fig. 3) between visual and olfactory attention (two-way mixed ANOVA with Greenhouse-Geisser adjustment: time: *F*(2,188) = 37.26, *P* = 8.46e-12; time × modality: *F*(2,188) = 0.81, *P* = 0.420). (**d**) Comparisons in firing rate of neuron #5 in the time windows of DA1, DA2, and DA3 (see Fig. 3) between visual and olfactory attention (two-way mixed ANOVA with Greenhouse-Geisser adjustment: time: *F*(2,188) = 12.20, *P* = 1.84e-5; time × modality: *F*(2,188) = 0.95, *P* = 0.384). (**e**) Trend of normalized population activities of attention-related activated neurons in correct trials aligned to the time of trigger onset in visual and olfactory attention (n = 9, proportion = 9.68%). (**f**) Comparisons in normalized firing rate (see Fig. 3e) of all attention-related activated neurons (same as **e**) in the time windows of DA, BA, and AA between visual and olfactory attention (two-way ANOVA with Greenhouse-Geisser adjustment: modality: *F*(1,8) = 0.14, *P* = 0.719; time: *F*(2,16) = 12.59, *P* = 6.21e-4; modality × time: *F*(2,16) = 1.41, *P* = 0.273). (**g**) Comparisons in normalized firing rate (see Fig. 3e) of all attention-related activated neurons (same as **e**) in the time windows of DA1, DA2, and DA3 between visual and olfactory attention (two-way ANOVA with Greenhouse-Geisser adjustment: modality: *F*(1,8) = 0.01, *P* = 0.925; time: *F*(2,16) = 0.10, *P* = 0.808; modality × time: *F*(2,16) = 1.62, *P* = 0.230). The statistic results showed in panel c,d,f, and g were from post hoc simple effect analyses (MANOVA).

To determine whether visual and olfactory attention tasks share common mechanisms in the ACC, we undertook tasks of both visual and olfactory sustained attention by training rats to perform blocks of both visual and olfactory tasks while we recorded ACC neuronal activity. We observed no significant differences in behavioral performance between modalities (Fig. 4a, accurate: *t*(10) = 0.33, *P* = 0.750; premature: *t*(10) = 0.35, *P* = 0.733; omission: *t*(10) = 1.31, *P* = 0.220; retrigger: *t*(10) = 1.56, *P* = 0.149), and no significant differences were seen in persistent activity of neurons activated in attention period between modalities (Figs 4e–g and 4b–d shows an example of a neuron that belongs to the type shown in Figure S7c).

### Trial-to-trial variability in neuronal activity correlates with sustainment of attention regardless of modality

**Figure 5:**
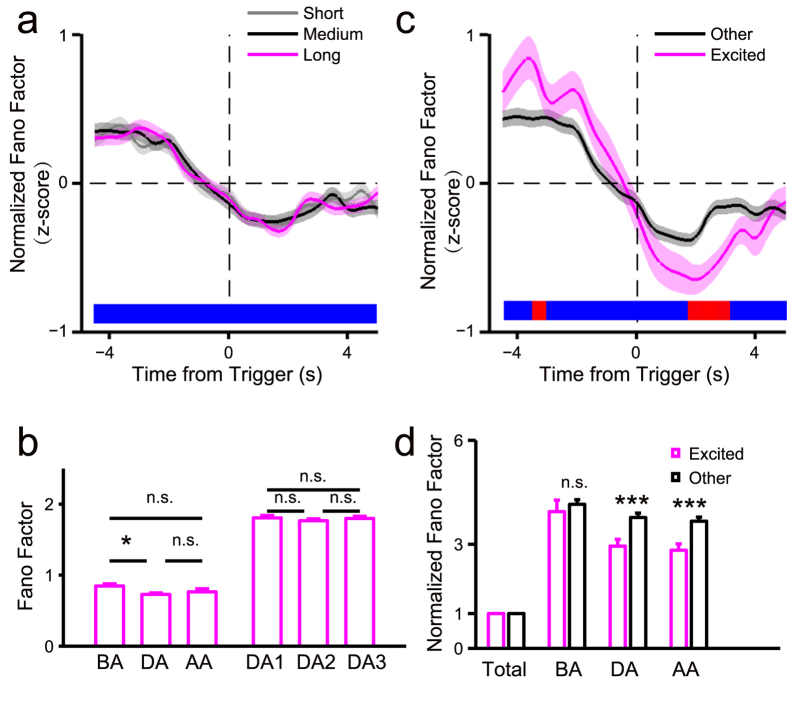
Fano factor of the recorded neurons in visual task was correlated with sustained attention. The data were the same as data in Fig. 3: rat no. = 4, all recorded neuron no. = 351, excited neuron no. = 46. (**a**) Trend of normalized fano factor of all recorded neurons in correct trials at the three TSI values aligned to the time from trigger. (**b**) Comparisons in fano factor among different time windows (see Fig. 3 for definitions of time windows) (one-way ANOVA: BA, DA, and AA: *F*(2,1044) = 3.51, *P* = 0.030; DA1, DA2, and DA3: *F*(2,1044) = 0.47, *P* = 0.627). The statistic results showed in this panel were from post hoc multiple comparisons of one-way ANOVA with Bonferroni adjustment. (**c**) Trend of normalized fano factor for excited and other neurons in correct trials aligned to the time from trigger. (**d**) Comparisons in normalized fano factor (normalized to fano factor of the total time window) in time windows of BA, DA, or AA between excited and other neurons (two-way mixed ANOVA with Greenhouse-Geisser adjustment: time: *F*(2,698) = 26.35, *P* = 1.90e-11; time × neuron type: *F*(2,698) = 6.35, *P* = 0.002). The statistic results showed in this panel were from post hoc simple effect analyses (MANOVA).

We examined trial-to-trial variability of neuronal activity using the Fano factor, as previous studies indicate that it is useful to evaluate differences between attention and non-attention states^23^,^24^. For all neurons recorded in the visual task, including those not activated or suppressed during attention, the Fano factor during sustained attention was reduced relative to that in the pre-attention time window (Fig. 5a,b, Figure S14a–d). Furthermore, the Fano factor remained constant during sustained attention, and we observed no significant differences among early, middle and late segments of the attention period (Fig. 5b). Further comparisons between attention-related excited neurons and other neurons showed significant differences during and after attention but not before (Fig. 5c,d).

The Fano factor of neuronal activity followed the same pattern seen in olfactory and visual tasks (Figure S14e–h, Figure S15): namely, trial-to-trial variability of recorded neurons decreased with sustained attention and remained low in the attention period, and attention-related neurons showed lower trial-to-trial variability during the attention period relative to other neurons, regardless of modality.

### Auditory distractors do not perturb persistent neuronal activity in ACC

**Figure 6:**
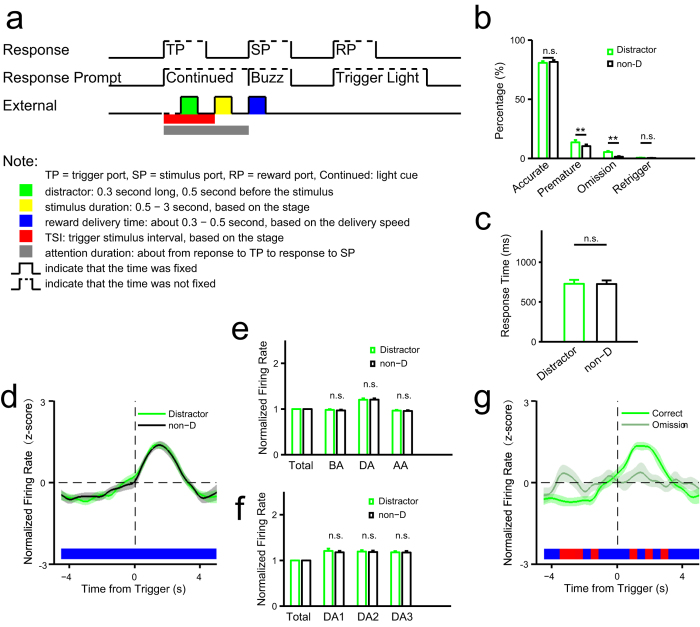
Effect of an auditory distractor on sustained attention. (**a**) Schematic of events in one trial (for comparison see Fig. 1d). A 0.3-second auditory distractor was delivered 0.5 second before stimulus delivery. (**b**) Comparisons of behavioral performance between trials with and without distractor (rat no. = 23, non-D: trials without distractor) (accurate: *t*(22) = 0.85, *P* = 0.407; premature: *t*(22) = 3.60, *P* = 0.0016; omission: *t*(22) = 3.44, *P* = 0.0024; retrigger: *t*(22) = 0.79, *P* = 0.441). (**c**) Comparisons of reaction time between trials with and without distractor (*t*(22) = 0.17, *P* = 0.867). (**d–g**) Electrophysiological data (rat no. = 4). (**d–f**) Comparisons between trials with and without distractor for all attention-related activated neurons (n = 19). (**d**) Trend of normalized firing rate in correct trials aligned to the time from trigger. (**e**) Comparisons in normalized firing rate (see Fig. 3e) in the three time windows BA, DA and AA (see Fig. 3c) between distractor and non-distractor trials (two-way ANOVA with Greenhouse-Geisser adjustment: distractor: *F*(1,18) = 1.81, *P* = 0.195; time: *F*(2,36) = 53.43, *P* = 5.92e-11; distractor × time: *F*(2,36) = 0.22, *P* = 0.801). (**f**) Comparisons in normalized firing rate in the three time windows DA1, DA2 and DA3 (see Fig. 3c) between distractor and non-distractor trials (two-way ANOVA with Greenhouse-Geisser adjustment: distractor: *F*(1,18) = 0.03, *P* = 0.868; time: *F*(2,36) = 3.08, *P* = 0.072; distractor × time: *F*(2,36) = 0.77, *P* = 0.465). (**g**) Trend of normalized firing rate of attention-related activated neurons (n = 13) in correct and omission trials in distractor condition with omission trials. The statistic results showed in panel b and c were from *t* tests. The statistic results showed in panel e and f were from post hoc simple effect analyses (MANOVA).

Our findings suggest that mechanisms underlying attention in different sensory modalities share common neuronal mechanisms in ACC. To further investigate the effect of other modalities on sustained attention, we introduced a transient auditory tone as a distractor prior to stimulus delivery to potentially perturb sustained attention and/or neuronal activity (Fig. 6a). In the test session, half of the trials employed random presentation of the distractor tone, enabling us to compare performance with and without it. We observed no differences in accuracy or response time between the presence and absence of the tone (Fig. 6b,c). However, the presence of the distractor resulted in more trials with premature responses and omitted trials (Fig. 6b), suggesting that sustained attention can be disturbed by an auditory signal without altering accuracy in finding the stimulus-delivery port. We then compared neuronal activity in distractor and non-distractor correct trials (Fig. 6d–f) and observed no significant differences in firing rate of activated neurons during the period of sustained attention. Since the presence of the distractor increased the proportion of omitted trials, we compared neuronal activity between correct and omitted trials. The results showed that distractor-associated omission reduced attention-related neuronal activity (Fig. 6g).

### External visual input does not drive persistent neuronal activity in the ACC

**Figure 7:**
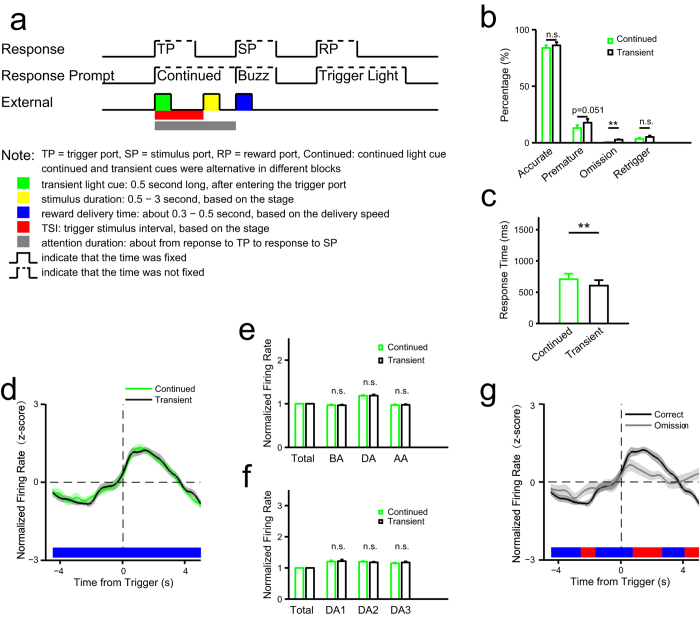
Effect of external visual input on sustained attention. (**a**) Schematic of events in one trial (for comparison see Fig. 1d). Continued cue, the cue was presented until the rat responded to the stimulus port; transient cue, 0.5 second long, both were presented when the animal entered the trigger port, and they were alternated in different blocks in each session. (**b**) Comparisons of behavioral performance between continued and transient cue blocks (accurate: *t*(12) = 0.97, *P* = 0.349; premature: *t*(12) = 2.17, *P* = 0.051; omission: *t*(12) = 3.95, *P* = 0.0019; retrigger: *t*(12) = 1.24, *P* = 0.239). (**c**) Comparisons of reaction time between continued and transient cue blocks (*t*(12) = 4.03, *P* = 0.0017). (**d–g**) Electrophysiological data (rat no. = 3). (**d–f**) Comparisons in activities of all attention-related activated neurons (n = 19) between continued and transient cue blocks. (**d**) Trend of normalized firing rate in correct trials aligned to the time from trigger. (**e**) Comparisons in normalized firing rate (see Fig. 3e) in the three time windows BA, DA and AA (see Fig. 3c) between continued and transient cue blocks (two-way ANOVA with Greenhouse-Geisser adjustment: cue: *F*(1,18) = 0.20, *P* = 0.658; time: *F*(2,36) = 37.89, *P* = 2.11e-8; cue × time: *F*(2,36) = 0.18, *P* = 0.818). (**f**) Comparisons in normalized firing rate in the three time windows DA1, DA2 and DA3 (see Fig. 3c) between continued and transient cue blocks (two-way ANOVA with Greenhouse-Geisser adjustment: cue: *F*(1,18) = 0.23, *P* = 0.639; time: *F*(2,36) = 0.74, *P* = 0.418; cue × time: *F*(2,36) = 0.84, *P* = 0.400). (**g**) Trend of normalized firing rate of attention-related activated neurons (n = 19) in correct and omission trials in transient blocks with omission trials. The statistic results showed in panel b and c were from *t* tests. The statistic results showed in panel e and f were from post hoc simple effect analyses (MANOVA).

Previous studies showed that the ACC can process a visual signal^25^, suggesting that external visual input is a source of persistent neuronal activity during sustained attention. To distinguish potential internal and external sources, we manipulated the length of a visual cue of a successful trigger in well-trained rats. We alternatively presented either transient or continuous cues, in one of two blocks in each test session (Fig. 7a). If driven by external visual input, neuronal activity in the transient cue block should also be transient. We observed no significant difference in terms of accuracy and proportion of premature responses between transient and continued cue blocks (Fig. 7b). However, rats responded more rapidly in transient than in continuous cue blocks (Fig. 7c). Notably, the percentage of omitted trials in transient blocks was greater than that in continuous blocks (Fig. 7b). Thus, loss of the transient signal tended to trigger withdrawal of sustained attention on the stimulus delivery port. Moreover, we observed no significant difference in neuronal activity between continuous and transient blocks (Fig. 7d–f): persistent neuronal activity was sustained until rats responded, even if the cue associated with initiation of attention was transient, strongly suggesting that that neuronal activity is not driven by external visual input. Further comparison of correct and omitted trials revealed significant differences in neuronal activities during the attention period in correct trials (Fig. 7g).

## Discussion

In this study, we developed a task to assess sustained attention in rats in both visual and olfactory modalities based on the widely used 5CSRTT paradigm^20^. Then, using pharmacological lesion experiments, we employed it to assess function of ACC in sustained attention. We then recorded neuronal activity in ACC using multichannel extracellular recording techniques. The results showed that a group of ACC neurons was persistently activated during the period of attention, suggesting a correlation between ACC neuronal activity and sustainment of attention. The following results demonstrated this correlation. First, modality of attention target had minimal influence on neuronal activity. Second, although the presence distractors during the attention period resulted in a greater number of premature responses and omitted trials, we observed no significant difference in attention-related neuronal activity in terms of accuracy between the presence and absence of distractors (Fig. 6). Third, we demonstrated that persistent activity seen during sustained attention is not driven by sensory input (Fig. 7). Furthermore, trial-to-trial variability of population activities in the ACC decreased significantly within the attention period, especially in attention-related neurons (Fig. 5, Figure S15).

To clarify the correlation between ACC neuronal activity and sustained attention, we should also eliminate potential psychological interferences including reward expectation, motivation, and working memory. In the present task, reward expectation and motivation should be equivalent for correct and incorrect trials and for omitted and correct trials, since the reward amount does not change during testing. But the neuronal activity between correct and incorrect trials, as well as omitted and correct trials was significantly different (Figs 3f–h, 6g and 7g). Thus, it is unlikely that these differences were due to altered reward expectation and motivation but rather emerged from changes in the psychological state of attention. Also, tests of working memory usually employ different objects to memorize in each trial^26^. However, we did not train rats to memorize different objects in each trial but rather to recall the task rules. Such recall could not be sustained during the entire period of attention and differed in correct and incorrect trials. Thus, the present task likely eliminates potential interference by working memory. However, attention-related neurons may also be activated or suppressed in other contexts, suggesting that these neurons are active in other states in addition to attention. First, neurons inhibited during sustained attention were maximally activated at initiation of reward consumption (Figure S8f, Figure S12f), suggesting they function in the reward process, in accordance with previous findings that ACC activity is associated with reward processing^27^. Second, two of three types of activated attention-related neurons identified were activated or suppressed simultaneously with the trigger (Fig. 4a, Figure S7b,c), suggesting they function in response to the trigger or in initiating attention.

To our knowledge, ours is the first study to investigate potential effects of target modality on the neural basis of sustained attention. We observed that the same neurons participated in both visual and olfactory attention, suggesting a common ACC pathway functioning in sustained attention. These findings are consistent with the idea that the attention system enhances all sensory input processing^1^, although deployment of attention is distinct for different targets^28^,^29^. During sustained attention in our experimental paradigm, properties of the detected target shaped strategies to detect the stimulus port. For example, in the olfactory task, rats were required to put their nose near the delivery site to detect an olfactory stimulus, while rats could detect visual stimuli more rapidly (Figure S2b), likely because they did not need to evaluate stimulus ports one by one. Thus, distinct detection strategies may underlie differences in response time between modalities or differences in behavioral performance or neuronal activity. However, we found that activity of certain types of ACC neurons did not differ between modalities (Fig. 4), suggestive of common mechanisms related to attention.

It should be noted that our lesion results differed in some respects from previous studies. First, decreased accuracy resulting from the ACC lesion was temporary (Fig. 2), although comparable lesion studies of visual attention report long-term deficits in attention^12^,^13^. Second, here the proportion of premature responses increased following lesioning, but a similar study reported an increase in the proportion of premature responses following lesion of the orbital frontal cortex (OFC) and infralimbic cortex (IL) but not after ACC lesion^12^. These differences might be due to differences in the extent or location of lesioned sites: sites in previous studies (AP + 2.2 ~ + 3.2) were approximately two millimeters anterior to lesion sites employed here (AP 0 ~ + 1). Also our experimental design differed from previous studies: ours is the first to use a trigger aperture to control the period of sustained attention.

It is noteworthy that the decrease in the Fano factor of neuronal activity started about 2 sec before the trigger. Here, except for sustained attention, the event sequence and locomotion of animals were comparable across trials, which could also decrease trial-to-trial variability of neuronal activity and lead to a decrease in the Fano factor before the trigger. However, the fact that the Fano factor decreased more significantly after rather than before the trigger and then differed significantly between attention-related activated neurons and other neurons suggests that sustained attention correlates with a decrease in the Fano factor.

ACC function is extensive and includes affective processing (such as regulation of emotion^30^), reward processing^27^, and representation of both physical^31^,^32^ and social^33^ pain. The ACC also participates in cognitive control through conflict monitoring^10^,^34^,^35^ and error detection^36^,^37^,^38^,^39^. Bush *et al*. proposed that the ACC is divided into dorsal cognitive and ventral affective subdivisions that interact with each other^30^, potentially accounting for the structure’s complex function. Our results, along with previous studies of ACC function in attention, suggest a slightly different perspective, namely, that both affective and cognitive processing consumes sustained attention, leading to persistent neuronal activity in ACC as a critical part of the attention system.

We analyzed neuronal activity in the ACC only. However, others note that multiple brain regions function in sustained attention^1^,^8^. The ACC is clearly an important component of this network that cooperates with other brain regions to enable sustained attention. In fact, a pattern of persistent neuronal activity has been observed in medial prefrontal cortex in tasks of attention^11^,^21^ and waiting^40^, and similar persistent OFC activity is seen in a waiting task^41^. Also, although our study showed comparable ACC activation in visual and olfactory attention, brain networks underlying sustained attention may differ for particular modalities. Therefore, understanding of mechanisms underlying sustained attention requires investigation of attention-related activity in the entire network across modalities.

## Methods

### Ethics statement

All experiments were carried out according to the protocol approved by the Animal Care and Use Committee of the Institute of Neuroscience, Chinese Academy of Sciences, and adhered to guidelines of the Ministry of Science and Technology of the People’s Republic of China for care and use of laboratory animals. All surgeries were performed under anesthesia, and efforts were made to minimize the number of animals and their suffering.

### Animals

In total, 49 male Sprague-Dawley rats (weighing 250 g at the start of training) were used in the present experiments: 34 in the visual task and 26 in the olfactory task (13 in both visual and olfactory tasks). Rats were housed in pairs in an individual ventilation cage (IVC) system on a 12-hour light/dark cycle with training and test sessions during the light cycle. During training and test sessions, limited water (~20 ml per day per rat) was delivered to the home cage after the task. One rat could take part in both visual and olfactory tasks.

### Test apparatus

The dimensions of the custom-made behavioral chamber were 30 × 26 × 28 cm^3^. All six sides were aluminum and when connected functioned like a faraday cage. The top side was chimney-shaped (24 × 16 × 14 cm^3^) to prevent rats from escaping and enable extracellular recording without loss of the faraday shield effect. The behavior chamber was placed in a soundproof iron box. Odor (valve: V290-4E, SYM Corp., South Korea) and reward (valve: USB2-M5-2, CKD Corp., Japan) delivery systems were placed outside the box to reduce noise pollution. Plastic pipes (inner diameter, 2.5 mm; SANG-A PNEUMATIC CO., LTD., South Korea) were used to deliver odors, air and reward water.

The task panel had one trigger aperture and three stimulus apertures (Fig. 1a). The vertical distance between trigger and stimulus apertures was 2 cm, and the horizontal distance was 4 cm. Aperture depth was 2.5 cm. Light emitting diodes delivering transient visual stimuli were located at the center of the aperture base (Fig. 1b). The odor delivery site was at the bottom of the stimulus apertures (see Fig. 1b; inner diameter of the pipe orifice was 1.5 mm). The infrared emitter and receiver used to detect the animal’s snout were located in the apertures and 0.6 cm from the aperture entrance.

Valves were used to control delivery of the odor isoamyl acetate (IAA), air and reward water. To deliver olfactory stimuli, air passed through mineral oil containing dissolved IAA (1:1000), and IAA was delivered to the delivery site 100 milliseconds later after valves were opened (Fig. 1c; odor concentration was measured by a photoionization detector, MiniPID, Ion Science INC, USA). When valves were closed, air passed through the mineral oil at a flow rate of ~0.8 liter per minute (flowmeter: LZB-3WB, ZHENXING Corp., Yuyao, Zhejiang, China). For reward delivery, a water port was placed at the opposite site of the task panel to control direction bias in stimulus detection. The reward delivery site was at the bottom of the water port and below the infrared device used to detect reward consumption. The liquid level was ~25 cm above the water port to control flow rate. Delivery lasted 150 milliseconds to ensure receipt of ~0.05 ml water for each correct trial.

### Behavioral training and tests

Software used for behavioral training and test was from Anilab Software and Instruments Co., Ltd (Ningbo, Zhejiang, China). Rats were individually trained for one 30-min session daily. In that procedure, rats were free to feed but water-restricted. Each rat consumed 20 ml water daily after training or test sessions. Water used as reward during training contained saccharin (dissolved 5:10000) to increase motivation. In visual and olfactory tasks, rats were trained to poke the trigger aperture with their nose to promote transient stimulus delivery from one of three stimulus apertures after a random time interval (TSI). To obtain the reward rats had to detect the location of a forthcoming transient stimulus and poke the aperture that had presented the stimulus (Fig. 1a,d,e, Supplementary video S1 and S2).

The initial training procedure (Figure S1) was used to train the movement sequence. After rats completed at least 80 trials for 2 consecutive sessions, training moved to stage 2 in which only one aperture delivered a stimulus. From then on, as TSI increased and time of stimulus presentation decreased, tasks become more difficult as an attention state was initiated (modified as a 3-choice test from the 5CSRTT^20^; see Totah and colleagues^11^ and Jacobson and colleagues^42^). Once the number of correct trials exceeded 50, accuracy (percentage of correct responses relative to correct plus incorrect trials) usually was greater than 80%, the proportion of both premature responses and repeated-trigger responses was less than 20%, and the proportion of omissions was less than 10% for 2 consecutive sessions. At that point the training moved to next stage.

Due to differences in difficulty of detecting visual and olfactory stimuli, the highest training stage was set differently for the two tasks. Stimulus duration was 0.5 sec in the highest stage of the visual task, and 1 sec in the olfactory task. Thus, there were seven stages in training of visual attention and six in training of olfactory attention. In the test task for both modalities, the TSI was 1, 1.5, or 2 seconds randomly applied in a single session. Rats passing the highest training stage were selected to perform the test task. Rats that met progression criteria in test tasks were used in lesion or physiological studies.

### Surgery

For electrophysiological studies, rats were surgically implanted with a silicon-based electrode (NeuroNexus, USA, A4 × 2-tet-5 mm-150-200-121-CM32, Figure S4a) or a custom made nickel-cadmium (KANTHAL Precision Technology, Palm Coast, FL, USA, diameter: 0.0005 in, impedance: ~500 kΩ)-based microdrive tetrode in the ACC of the right hemisphere (Figure S4b–d) under pentobarbital sodium (80 mg/kg, i.p.) anesthesia. Atropine sulfate (0.05 mg/kg, i.p.) was also administered to prevent breathing difficulties. Gentamycin (5 mg/kg, i.p.) and hexadecadrol (1 mg/kg, i.p.) were injected after surgery to prevent infection. Acrylic dental cement with skull screws was used to stabilize the electrode assembly. The electrode site was checked after recording sessions using a histological method.

For lesioning, we used the same anesthesia and infection prevention procedures used in electrophysiological studies. The experimental group received bilateral injection of ibotenic acid (IBO, 10 mg/ml, Sigma, USA) in the ACC (600 nanoliters on each side, Fig. 2a–c); the control group received same amount of normal saline bilaterally in the ACC. IBO lesion sites were checked after behavioral tests using a histological method.

### Data acquisition and analyses

Physiological test sessions were initiated 5 to 7 days after surgery during which time water was not restricted. The time of test session was not limited and determined by the rat’s performance (usually less than 60 min) in order to collect as many trials as possible. Motorized Commutators (Plexon Inc., Dallas, TX, USA) were used to prevent wire entanglement by walking rats. Neuronal activity recorded from microelectrode arrays was acquired using an OmniPlex TM data acquisition system (Plexon Inc.). Digitized signals were sampled at 40 kHz. Spike waveforms were collected using a simple threshold with a length of 1.2 milliseconds. Time-stamps acquired from the behavioral system were sent to the physiological system to synchronize neuronal and behavioral data. Each rat was tested in one session per day.

Most data processing was carried out using MATLAB. Offline single units were manually sorted using MClust Spike Sorting Toolbox (version 3.5)^43^. Waveform features used for sorting were peak amplitude, valley amplitude, energy, and principle components. A unit was verified as isolated based on three criteria: first, refractory period should be more than 1 millisecond showed by auto-correlogram (e.g., Figure S5); second, isolation distance should be more than 15, L-ratio should be less than 0.2; third, waveforms during entire recording session should be stable. Peri-event time histograms (PETHs) were calculated with a 500 millisecond interval for each condition. All PETHs shown in the results were normalized (transformed into z-scores). Comparisons between conditions were made in specific time windows, such as attention duration. For each time window, the firing rate was normalized with mean firing rate of the total time window (for reference of this simple normalization method for small samples see Murakami *et al*.^44^ when they normalized movement time in patient and impatient conditions and Zhang *et al*.^45^ when they normalized postsynaptic currents and orientation tuning of neuron activities). To characterize trial-to-trial variability of neuronal activity during sustained attention, the Fano factor^46^ was computed with a bin of 500 milliseconds.

The reported trends of measured values such as normalized firing rate (e.g., Fig. 3d) and normalized Fano factor (e.g., Fig. 5a) were shown with color bar that is thermograph of *P* value of one-way ANOVA used to compare among the conditions over time (Red: *P* ≤ 0.05; Blue: *P* > 0.05, time interval: 500 ms). The grey transparent shadow around these trends indicates S.E.M. of the measured values. All the statistical data that we showed were mean ± S.E.M. For the statistical results in all figures the symbol *** indicates *P *≤* *0.001, ** indicates *P *≤* *0.01, * indicates *P *≤* *0.05, and n.s. = not significant.

### Histology

Histological examination was performed to confirm recording or lesion locations. In physiological studies, electric current (50 μA, 10 s) was used to lesion brain tissue around electrode sites. Tetrode sites (Figure S4) were deduced from the lesion center. After the electrical lesion, rats were anesthetized with pentobarbital sodium (80 mg/kg, i.p.), and their brains were removed after perfusion with paraformaldehyde. Brains were sectioned in 60 μm coronal sections, and slices were stained with Nissl to identify the lesion site. Histological methods used for lesion studies were comparable to those used for physiological studies, except there was no electric lesion.

## Additional Information

**How to cite this article:** Wu, D. *et al*. Persistent Neuronal Activity in Anterior Cingulate Cortex Correlates with Sustained Attention in Rats Regardless of Sensory Modality. *Sci. Rep.* **7**, 43101; doi: 10.1038/srep43101 (2017).

**Publisher's note:** Springer Nature remains neutral with regard to jurisdictional claims in published maps and institutional affiliations.

## Supplementary Material

Supplementary Video S1

Supplementary Video S2

Supplemental Information

## References

[b1] Sarter M., Givens B. & Bruno J. P. The cognitive neuroscience of sustained attention: where top-down meets bottom-up. Brain research. Brain research reviews 35, 146–160 (2001). 11336780 10.1016/s0165-0173(01)00044-3

[b2] Petersen S. E. & Posner M. I. The attention system of the human brain: 20 years after. Annual review of neuroscience 35, 73–89, doi: 10.1146/annurev-neuro-062111-150525 (2012). PMC3413263 22524787

[b3] Kerns J. G. . Anterior cingulate conflict monitoring and adjustments in control. Science 303, 1023–1026, doi: 10.1126/science.1089910 (2004). 14963333

[b4] Weissman D. H., Gopalakrishnan A., Hazlett C. J. & Woldorff M. G. Dorsal anterior cingulate cortex resolves conflict from distracting stimuli by boosting attention toward relevant events. Cerebral cortex 15, 229–237, doi: 10.1093/cercor/bhh125 (2005). 15238434

[b5] Roelofs A., van Turennout M. & Coles M. G. Anterior cingulate cortex activity can be independent of response conflict in Stroop-like tasks. Proceedings of the National Academy of Sciences of the United States of America 103, 13884–13889, doi: 10.1073/pnas.0606265103 (2006). 16954195 PMC1564252

[b6] Seidman L. J. . Dorsolateral prefrontal and anterior cingulate cortex volumetric abnormalities in adults with attention-deficit/hyperactivity disorder identified by magnetic resonance imaging. Biological psychiatry 60, 1071–1080, doi: 10.1016/j.biopsych.2006.04.031 (2006). 16876137

[b7] Sheth S. A. . Human dorsal anterior cingulate cortex neurons mediate ongoing behavioural adaptation. Nature 488, 218–221, doi: 10.1038/nature11239 (2012). 22722841 PMC3416924

[b8] Nagai Y. . Brain activity relating to the contingent negative variation: an fMRI investigation. NeuroImage 21, 1232–1241, doi: 10.1016/j.neuroimage.2003.10.036 (2004). 15050551

[b9] Isomura Y., Ito Y., Akazawa T., Nambu A. & Takada M. Neural coding of “attention for action” and “response selection” in primate anterior cingulate cortex. The Journal of neuroscience: the official journal of the Society for Neuroscience 23, 8002–8012 (2003). 12954861 10.1523/JNEUROSCI.23-22-08002.2003 PMC6740492

[b10] Ebitz R. B. & Platt M. L. Neuronal activity in primate dorsal anterior cingulate cortex signals task conflict and predicts adjustments in pupil-linked arousal. Neuron 85, 628–640, doi: 10.1016/j.neuron.2014.12.053 (2015). 25654259 PMC4319115

[b11] Totah N. K., Kim Y. B., Homayoun H. & Moghaddam B. Anterior cingulate neurons represent errors and preparatory attention within the same behavioral sequence. The Journal of neuroscience: the official journal of the Society for Neuroscience 29, 6418–6426, doi: 10.1523/JNEUROSCI.1142-09.2009 (2009). 19458213 PMC2730728

[b12] Chudasama Y. . Dissociable aspects of performance on the 5-choice serial reaction time task following lesions of the dorsal anterior cingulate, infralimbic and orbitofrontal cortex in the rat: differential effects on selectivity, impulsivity and compulsivity. Behavioural brain research 146, 105–119 (2003). 14643464 10.1016/j.bbr.2003.09.020

[b13] Passetti F., Chudasama Y. & Robbins T. W. The frontal cortex of the rat and visual attentional performance: dissociable functions of distinct medial prefrontal subregions. Cerebral cortex 12, 1254–1268 (2002). 12427677 10.1093/cercor/12.12.1254

[b14] Botvinick M., Nystrom L. E., Fissell K., Carter C. S. & Cohen J. D. Conflict monitoring versus selection-for-action in anterior cingulate cortex. Nature 402, 179–181, doi: 10.1038/46035 (1999). 10647008

[b15] Weissman D. H., Roberts K. C., Visscher K. M. & Woldorff M. G. The neural bases of momentary lapses in attention. Nature neuroscience 9, 971–978, doi: 10.1038/nn1727 (2006). 16767087

[b16] Walter W. G., Cooper R., Aldridge V. J., McCallum W. C. & Winter A. L. Contingent Negative Variation: An Electric Sign of Sensorimotor Association and Expectancy in the Human Brain. Nature 203, 380–384 (1964). 14197376 10.1038/203380a0

[b17] Tecce J. J. Contingent negative variation (CNV) and psychological processes in man. Psychological Bulletin 77, 73–108, doi: 10.1037/h0032177 (1972). 4621420

[b18] Cheal M. & Chastain G. Efficiency of visual selective attention is related to the type of target. Psychological research 66, 110–115 (2002). 12132113 10.1007/s00426-001-0083-0

[b19] Cheal M. L. & Lyon D. R. Benefits from attention depend on the target type in location-precued discrimination. Acta psychologica 81, 243–267 (1992). 1462787 10.1016/0001-6918(92)90020-e

[b20] Bari A., Dalley J. W. & Robbins T. W. The application of the 5-choice serial reaction time task for the assessment of visual attentional processes and impulse control in rats. Nature protocols 3, 759–767, doi: 10.1038/nprot.2008.41 (2008). 18451784

[b21] Kim H., Ahrlund-Richter S., Wang X., Deisseroth K. & Carlen M. Prefrontal Parvalbumin Neurons in Control of Attention. Cell 164, 208–218, doi: 10.1016/j.cell.2015.11.038 (2016). 26771492 PMC4715187

[b22] Donnelly N. A., Paulsen O., Robbins T. W. & Dalley J. W. Ramping single unit activity in the medial prefrontal cortex and ventral striatum reflects the onset of waiting but not imminent impulsive actions. The European journal of neuroscience 41, 1524–1537, doi: 10.1111/ejn.12895 (2015). 25892211 PMC4529742

[b23] Mitchell J. F., Sundberg K. A. & Reynolds J. H. Spatial attention decorrelates intrinsic activity fluctuations in macaque area V4. Neuron 63, 879–888, doi: 10.1016/j.neuron.2009.09.013 (2009). 19778515 PMC2765230

[b24] Cohen M. R. & Maunsell J. H. Attention improves performance primarily by reducing interneuronal correlations. Nature neuroscience 12, 1594–1600, doi: 10.1038/nn.2439 (2009). 19915566 PMC2820564

[b25] Laurienti P. J. . Cross-modal sensory processing in the anterior cingulate and medial prefrontal cortices. Human brain mapping 19, 213–223, doi: 10.1002/hbm.10112 (2003). 12874776 PMC6871917

[b26] Liu D. . Medial prefrontal activity during delay period contributes to learning of a working memory task. Science 346, 458–463, doi: 10.1126/science.1256573 (2014). 25342800

[b27] Hampton A. N. & O’Doherty J. P. Decoding the neural substrates of reward-related decision making with functional MRI. Proceedings of the National Academy of Sciences of the United States of America 104, 1377–1382, doi: 10.1073/pnas.0606297104 (2007). 17227855 PMC1783089

[b28] Eldar E., Cohen J. D. & Niv Y. The effects of neural gain on attention and learning. Nature neuroscience 16, 1146–1153, doi: 10.1038/nn.3428 (2013). 23770566 PMC3725201

[b29] Ebitz R. B., Pearson J. M. & Platt M. L. Pupil size and social vigilance in rhesus macaques. Frontiers in neuroscience 8, 100, doi: 10.3389/fnins.2014.00100 (2014). 24834026 PMC4018547

[b30] Bush G., Luu P. & Posner M. I. Cognitive and emotional influences in anterior cingulate cortex. Trends in cognitive sciences 4, 215–222 (2000). 10827444 10.1016/s1364-6613(00)01483-2

[b31] Rainville P., Duncan G. H., Price D. D., Carrier B. & Bushnell M. C. Pain affect encoded in human anterior cingulate but not somatosensory cortex. Science 277, 968–971 (1997). 9252330 10.1126/science.277.5328.968

[b32] Blom S. M., Pfister J. P., Santello M., Senn W. & Nevian T. Nerve injury-induced neuropathic pain causes disinhibition of the anterior cingulate cortex. The Journal of neuroscience: the official journal of the Society for Neuroscience 34, 5754–5764, doi: 10.1523/JNEUROSCI.3667-13.2014 (2014). 24760836 PMC6608297

[b33] Eisenberger N. I., Lieberman M. D. & Williams K. D. Does rejection hurt? An FMRI study of social exclusion. Science 302, 290–292, doi: 10.1126/science.1089134 (2003). 14551436

[b34] Botvinick M. M., Braver T. S., Barch D. M., Carter C. S. & Cohen J. D. Conflict monitoring and cognitive control. Psychological review 108, 624–652 (2001). 11488380 10.1037/0033-295x.108.3.624

[b35] Botvinick M. M., Cohen J. D. & Carter C. S. Conflict monitoring and anterior cingulate cortex: an update. Trends in cognitive sciences 8, 539–546, doi: 10.1016/j.tics.2004.10.003 (2004). 15556023

[b36] Ito S., Stuphorn V., Brown J. W. & Schall J. D. Performance monitoring by the anterior cingulate cortex during saccade countermanding. Science 302, 120–122, doi: 10.1126/science.1087847 (2003). 14526085

[b37] Carter C. S. . Anterior cingulate cortex, error detection, and the online monitoring of performance. Science 280, 747–749 (1998). 9563953 10.1126/science.280.5364.747

[b38] Kuwabara M., Mansouri F. A., Buckley M. J. & Tanaka K. Cognitive control functions of anterior cingulate cortex in macaque monkeys performing a Wisconsin Card Sorting Test analog. The Journal of neuroscience: the official journal of the Society for Neuroscience 34, 7531–7547, doi: 10.1523/JNEUROSCI.3405-13.2014 (2014). 24872558 PMC4035517

[b39] Brown J. W. & Braver T. S. Learned predictions of error likelihood in the anterior cingulate cortex. Science 307, 1118–1121, doi: 10.1126/science.1105783 (2005). 15718473

[b40] Xu M., Zhang S. Y., Dan Y. & Poo M. M. Representation of interval timing by temporally scalable firing patterns in rat prefrontal cortex. Proceedings of the National Academy of Sciences of the United States of America 111, 480–485, doi: 10.1073/pnas.1321314111 (2014). 24367075 PMC3890779

[b41] Xiao X., Deng H., Wei L., Huang Y. & Wang Z. Neural activity of orbitofrontal cortex contributes to control of waiting. The European journal of neuroscience 44, 2300–2313, doi: 10.1111/ejn.13320 (2016). 27336203

[b42] Jacobson T. K., Ho J. W., Kent B. W., Yang F. C. & Burwell R. D. Automated visual cognitive tasks for recording neural activity using a floor projection maze. Journal of visualized experiments: JoVE, e51316, doi: 10.3791/51316 (2014). 24638057 PMC4130232

[b43] Redish A. MClust: a spike-sorting toolbox. http://redishlab.neuroscience.umn.edu/MClust/MClust.html, 2011).

[b44] Murakami M., Vicente M. I., Costa G. M. & Mainen Z. F. Neural antecedents of self-initiated actions in secondary motor cortex. Nature neuroscience 17, 1574–1582, doi: 10.1038/nn.3826 (2014). 25262496

[b45] Zhang S. . Selective attention. Long-range and local circuits for top-down modulation of visual cortex processing. Science 345, 660–665, doi: 10.1126/science.1254126 (2014). 25104383 PMC5776147

[b46] Churchland M. M. . Stimulus onset quenches neural variability: a widespread cortical phenomenon. Nature neuroscience 13, 369–378, doi: 10.1038/nn.2501 (2010). 20173745 PMC2828350

