# Supplementary material for: Persistent Neuronal Activity in Anterior Cingulate Cortex Correlates with Sustained Attention in Rats Regardless of Sensory Modality
Source: Sci Rep. 2017 Feb 23;7:43101. doi: 10.1038/srep43101 (PMC5322335; doi:10.1038/srep43101)
Supplement: Supplemental Information [file srep43101-s3.doc]

**Persistent Neuronal Activity in Anterior Cingulate Cortex Correlates with Sustained Attention in Rats Regardless of Sensory Modality**

Dingcheng Wu^1^, Hanfei Deng^1, 2^, Xiong Xiao^1, 2^, Yanfang Zuo^1^, Jingjing Sun^1^, Zuoren Wang^1, CA^

^1^Institute of Neuroscience, CAS Center for Excellence in Brain Science, State Key Laboratory of Neuroscience, Shanghai Institutes for Biological Sciences, Chinese Academy of Sciences, Shanghai 200031, China

^2^Graduate School of University of Chinese Academy of Sciences, Beijing 100049, China

Postal address: A738 Life Science Building, 320 Yueyang Road, , Shanghai, China, 200031

^CA^ To whom correspondence should be addressed. Email: [zuorenwang@ion.ac.cn](mailto:zuorenwang@ion.ac.cn)

## SUPPLEMENTAL FIGURES


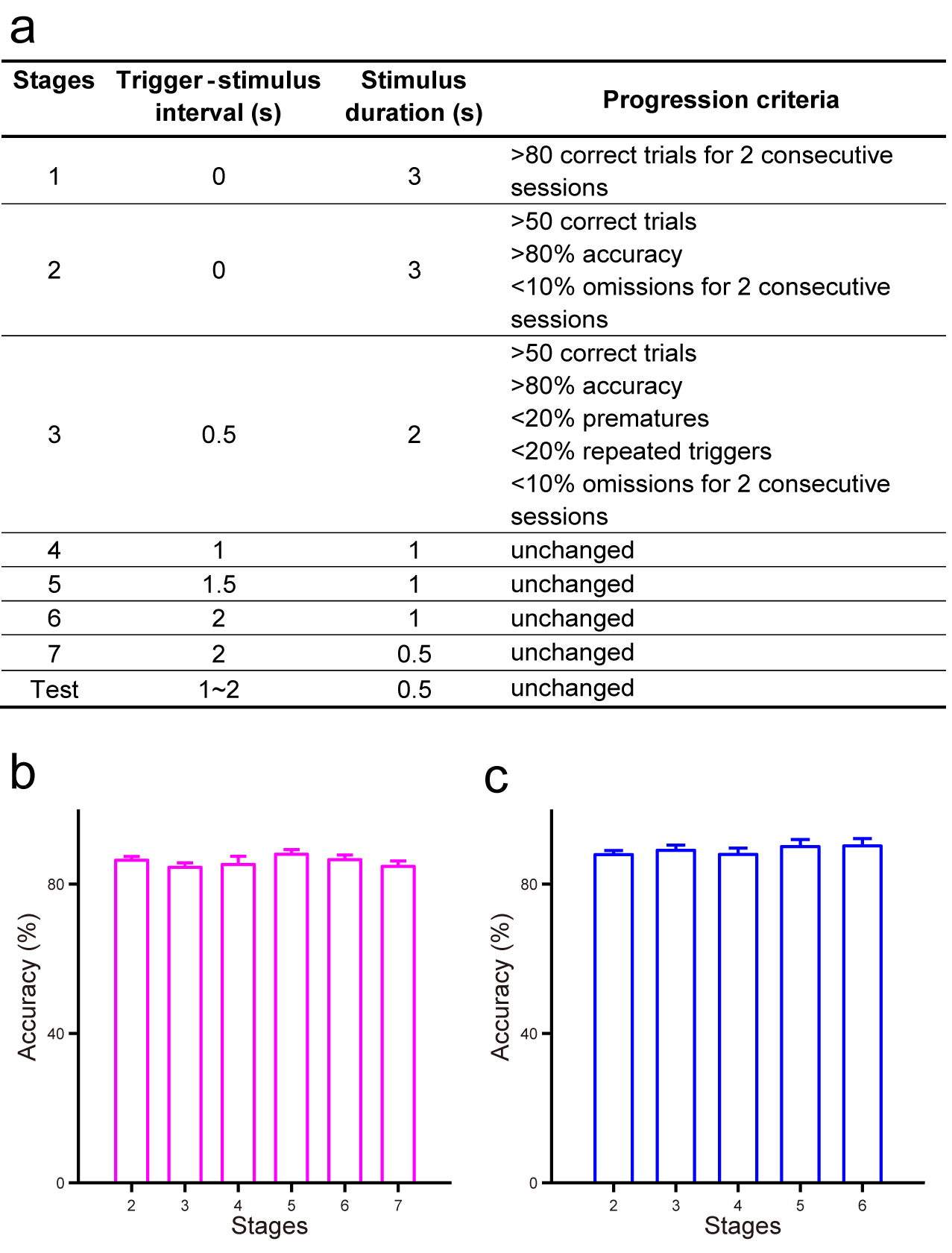


**Figure S1. Behavioral performance in each stage. (a) Training schedule for acquisition of sustained attention in visual and olfactory tasks. Note that the highest training stages were 7 for the visual task and 6 for the olfactory task.** (b) Accuracy in reaching criteria at each training stage in the visual task (n=17). (c) Accuracy in reaching criteria at each training stage in the olfactory task (n=14).


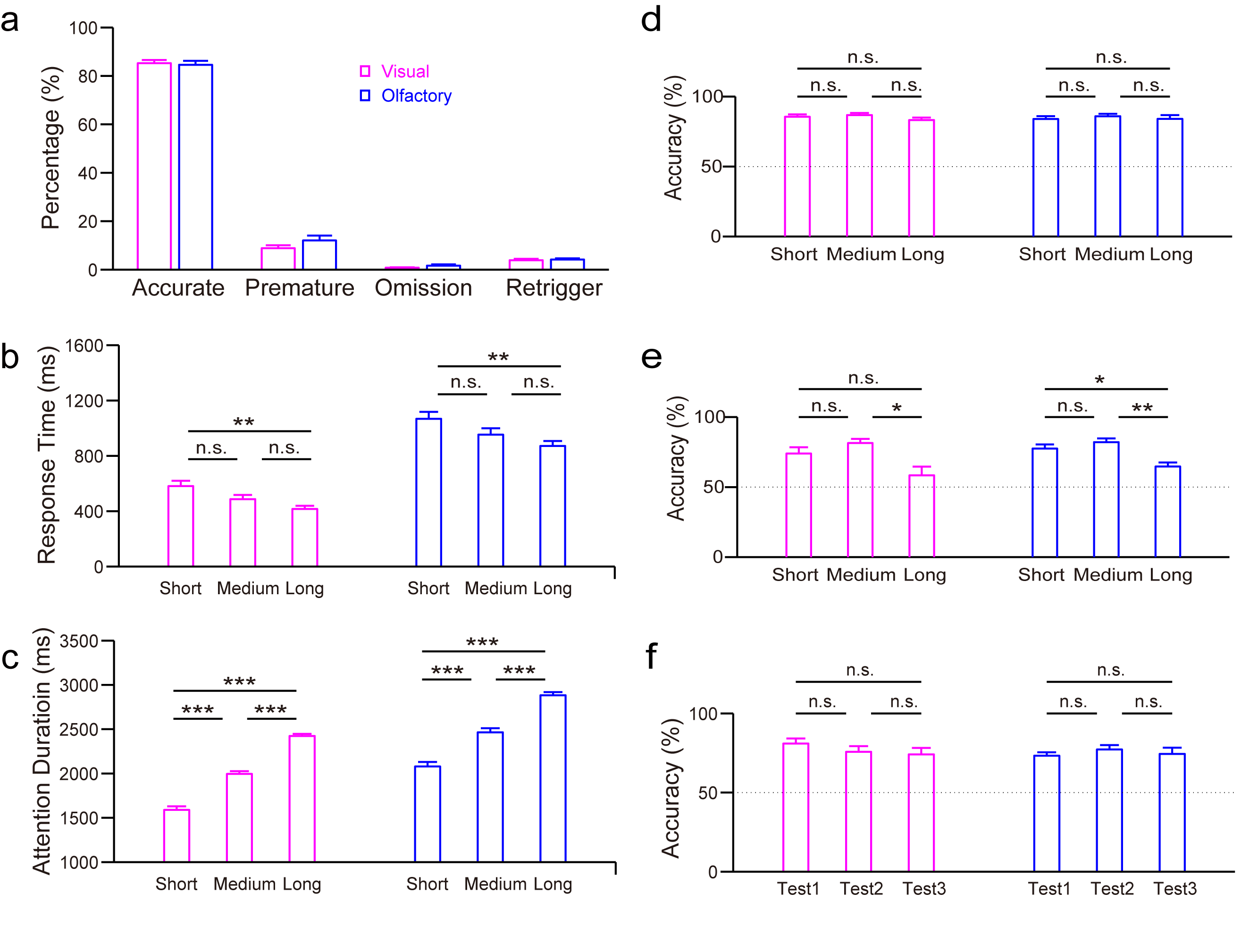


**Figure S2. Behavioral performance in the test task. (a) Main index of behavioral performance in test task** (Visual: n= 17, Olfactory: n = 15, TSIs: Short=1S, Medium=1.5S, and Long=2S, rats were same for b, c, and d)**. (b) The relationship between TSIs and response time in the test task** (one-way ANOVA: Visual: *F*(2,48) = 6.95, *P =* 0.002, Olfactory: *F*(2,42) = 4.93, *P =* 0.012)**. (c) The relationship between TSIs and attention duration in the test task** (one-way ANOVA: Visual: *F*(2,48) = 164.52, *P =* 3.29e-22, Olfactory: *F*(2,42) = 80.6, *P =* 4.19e-15)**.** (d) **The relationship between TSIs and accuracy in the test task.** TSIs: Short=1S, Medium=1.5S, and Long=2S (one-way ANOVA: Visual: *F*(2,48) = 1.18, *P =* 0.315, Olfactory: *F*(2,42) = 0.26, *P =* 0.770). (e) **The relationship between TSIs and accuracy in alternative test task.** TSIs: Short=0S, Medium=1.5S, Long=3S. (one-way ANOVA: Visual: *F*(2,21) = 3.81, *P =* 0.039, Olfactory: *F*(2,15) = 9.89, *P =* 0.0018). (f) **Memory tests of sustained attention** (one-way ANOVA: Visual: *F*(2,39) = 1.65, *P =* 0.206, Olfactory: *F*(2,33) = 0.74, *P =* 0.483)**.** The results of multiple comparisons showed in panel b-f were from post hoc of one-way ANOVA with Bonferroni adjustment. *** *P ≤* 0.001, ** *P ≤* 0.01, * *P ≤* 0.05. n.s. = not significant.


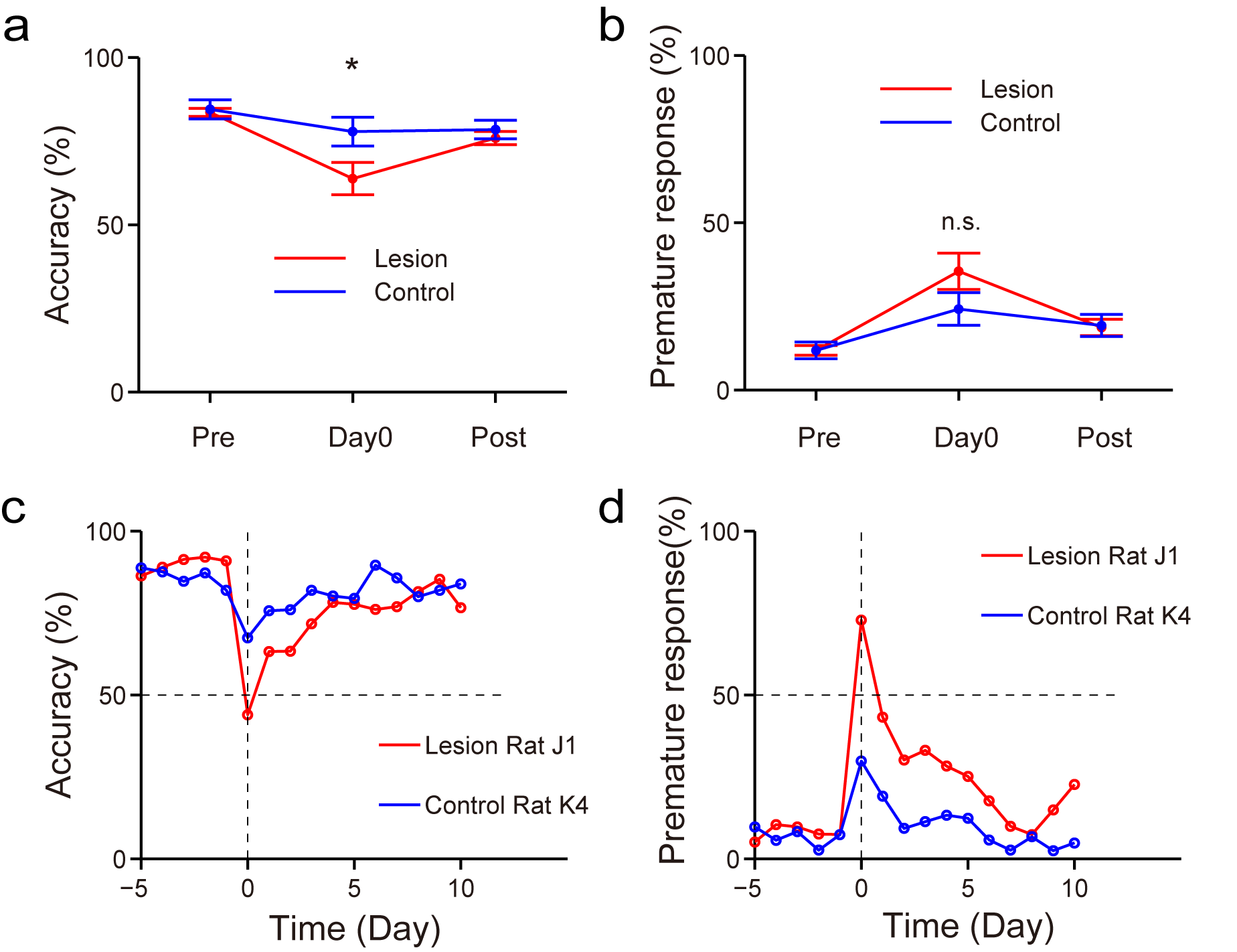


**Figure S3. Effects of ACC lesions on sustained attention.** (a) Comparisons between lesion and control group in accuracy for all rats in both visual and olfactory attention tasks. (Total: n = 20, lesion: n = 11, control: n = 9) (two-way mixed ANOVA with Greenhouse-Geisser adjustment: time: *F*(2,36) = 13.38, *P =* 0.001; time * lesion: *F*(2,36) = 3.95, *P =* 0.048). (b) Comparisons between lesion and control group in proportion of premature response for all rats (two-way mixed ANOVA with Greenhouse-Geisser adjustment: time: *F*(2,36) = 22.21, *P =* 4.92e-5; time * lesion: *F*(2,36) = 3.02, *P =* 0.090). (c) Typical rat example for lesion effect in accuracy. The accuracy was decreased by the ACC lesion and was gradually recovered in the training session after surgery. (d) Typical rat example for lesion effect in premature response. The premature proportion was increased by the ACC lesion and gradually decreased to the level before the surgery. Pre: averaged behavioral performance on five test days before surgery; Day0: behavioral performance on first post-surgery day; Post: averaged behavioral performance on five test days after first post-surgery day. The statistic results showed in panel a and b were from post hoc simple effect analyses (MANOVA). All the statistical data that we showed were mean ± S.E.M. *** *P ≤* 0.001, ** *P ≤* 0.01, * *P ≤* 0.05. n.s. = not significant.


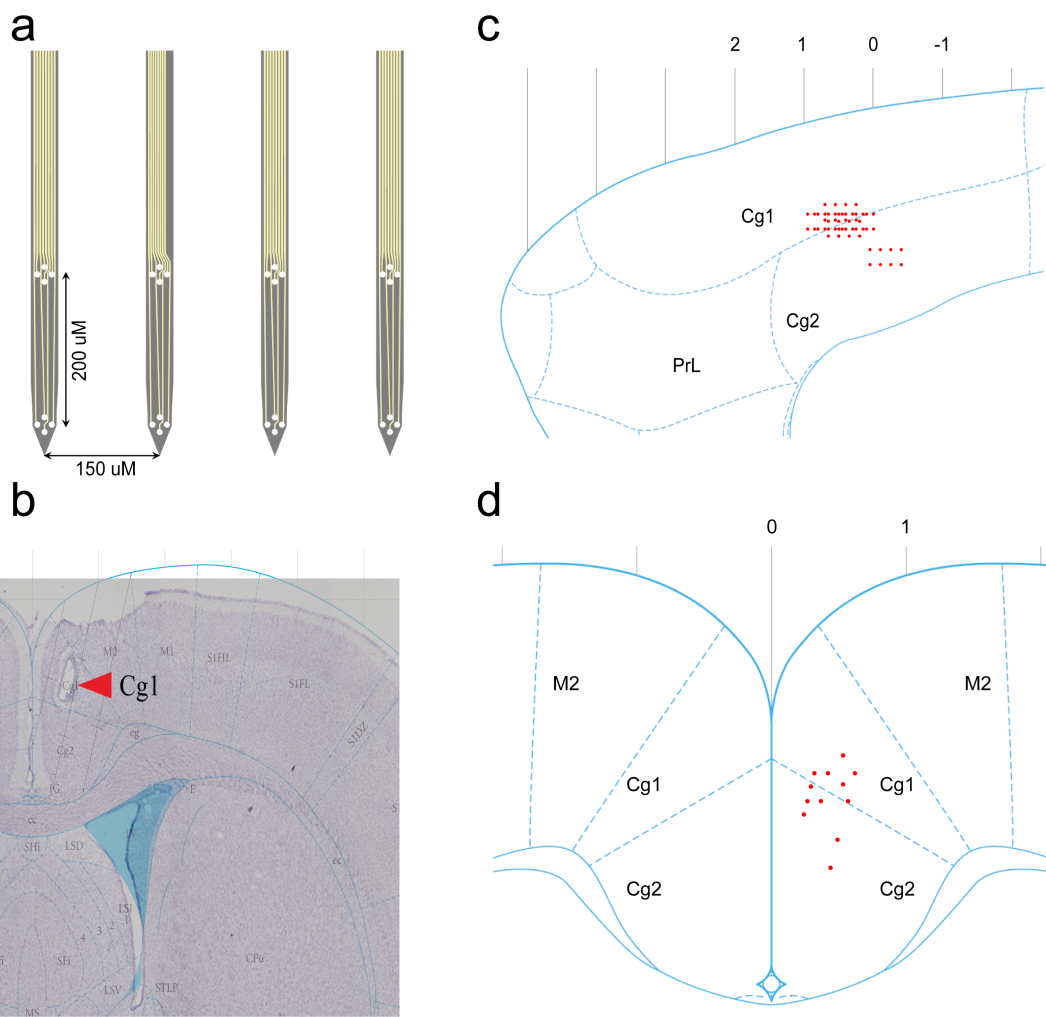


**Figure S4. Electrode sites.** (a) NeuroNexus silicon-based tetrode array used in this study. Each rat was implanted in the ACC with one array with eight tetrodes. (b) **Histochemistry results. Tissue section corresponded to comparable image from** Paxinos and Watson, 2007, which showed that approximate tetrode sites were in the ACC. (c) Tetrode sites targeting sagittal sections of brain (lateral 0.18 mm). (d) Tetrode sites targeting coronal sections of brain (bregma 0.48 mm). **Sites drawn in panels c and d were deduced from coordinates relative to bregma (coordinate: 0, 0, 0) recorded in the surgery procedure and confirmed by histochemistry, as shown in panel b. Sketches of rat brain were from** Paxinos and Watson, 2007.


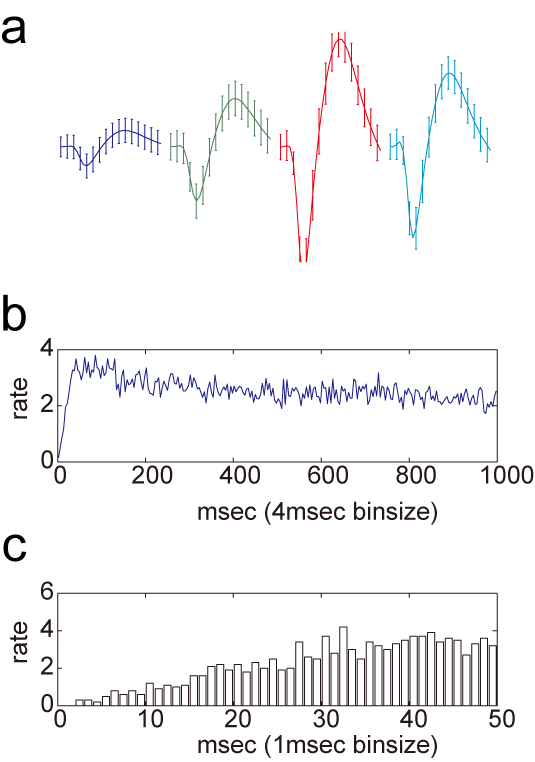


**Figure S5. Example of isolated unit sorted using MClust software.** (a) Spike waveforms of the unit recorded with four channels of a tetrode. (b) Auto-correlogram of the unit with 4 millisecond binsize. (c) Auto-correlogram of the unit with 1 millisecond binsize.


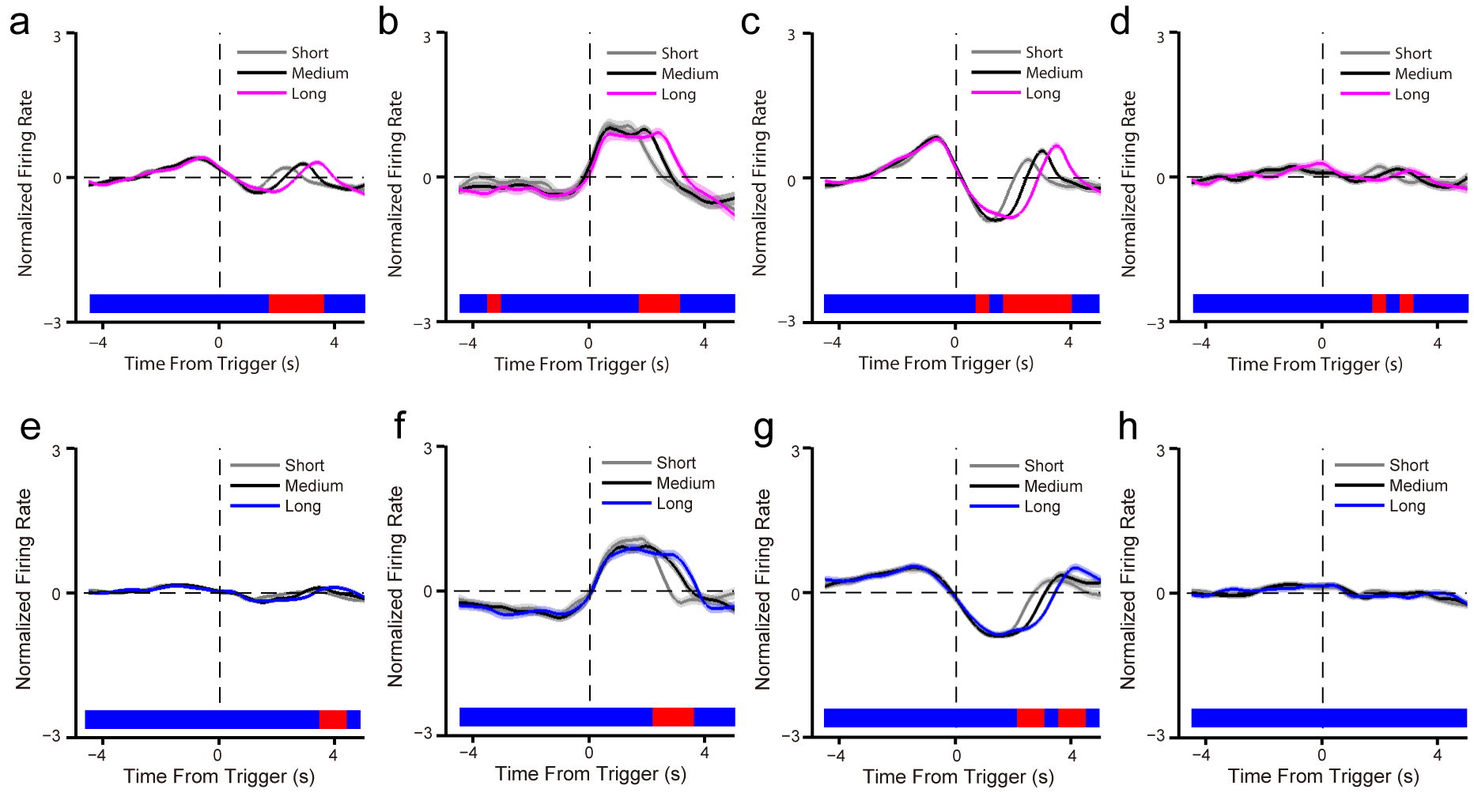


**Figure S6. Averaged trend of normalized firing rate for different types of neurons in correct trials in visual and olfactory attention task.** (a-d) Vision attention. (e-h) Olfactory attention. (a, e) All neurons. (b, f) Activated neurons during attention. (c, g) Suppressed neurons during attention. (d, h) Neurons whose activities did not show any significant changes during attention. The inset color bar in all panels beside x axis is thermograph of *P* value of one-way ANOVA used to compare in normalized firing rate (z-score) among the three TSI conditions showed in trends over time (Red: *P* ≤ 0.05; Blue: *P* > 0.05, time interval: 500 ms). The grey transparent shadow in all panels indicates S.E.M. of the y value.


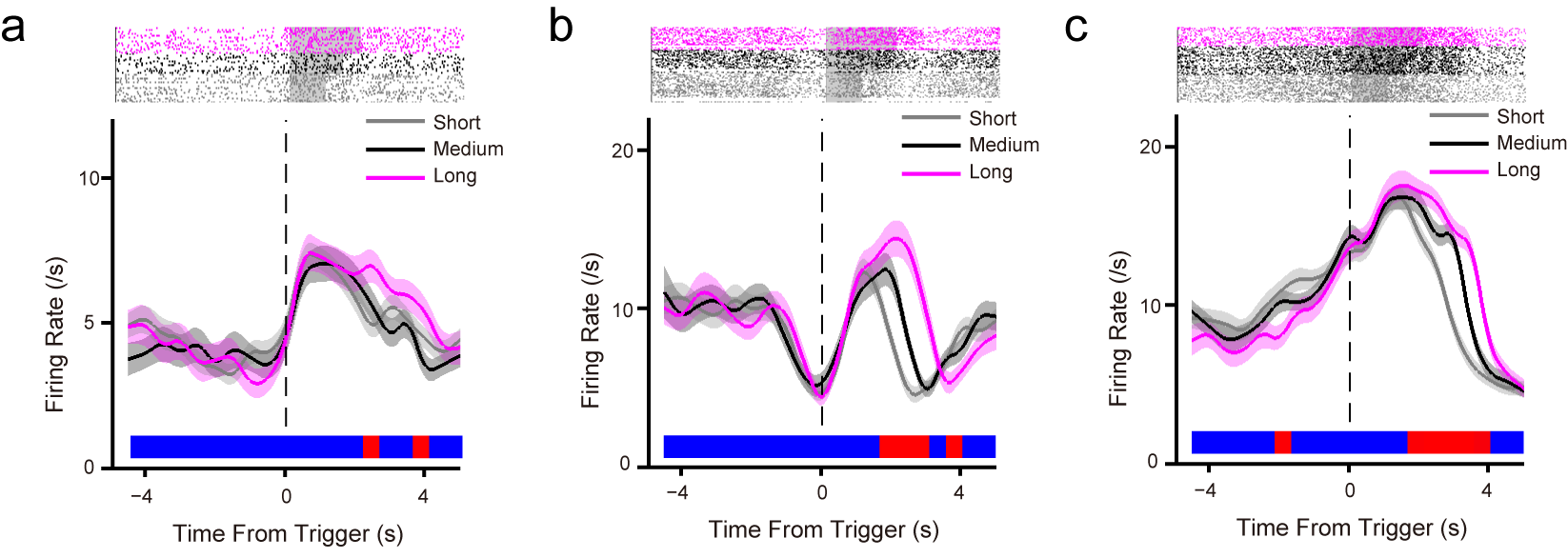


**Figure S7. Classification of attention-related activated neurons according to responses to trigger.** (a-c) Top: a raster plot of spikes at the 3 TSI values; grey transparent shadow indicates TSI time window; bottom: trend of firing rate at 3 TSI values aligned to the time from trigger in correct trials. (a) An example of neurons started being activated from trigger (neuron #157). (b) An example of neurons first inhibited at trigger, then activated after trigger (neuron #197). (c) An example of neurons started being activated before the trigger (neuron #233). The inset color bar in all panels beside x axis is thermograph of *P* value of one-way ANOVA used to compare in firing rate among the three TSI conditions showed in trends over time (Red: *P* ≤ 0.05; Blue: *P* > 0.05, time interval: 500 ms). The grey transparent shadow in all panels indicates S.E.M. of the y value.


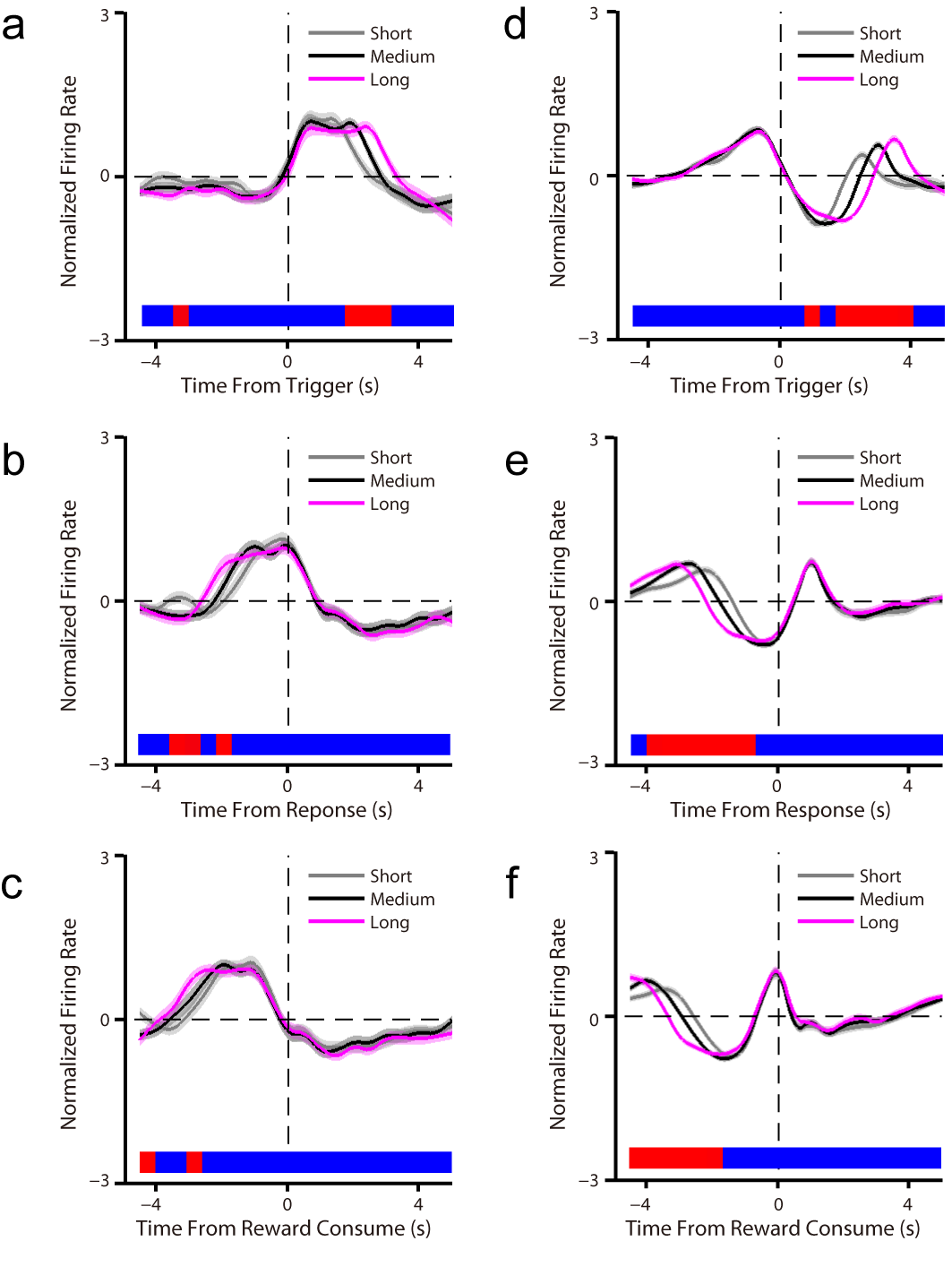


**Figure S8. Activities of two types of neurons during sustained visual attention.**

**(a-f)** Averaged trend of normalized firing rate of attention-related neurons in correct trials of the three TSI conditions (bin = 500ms). **(a-c) Activated neurons (n = 46). (d-f) Inhibited neurons (n = 158). (a, d)** Aligned to the time from trigger. **(b, e)** Aligned to the time from response. **(c, f)** Aligned to the time from reward consume. The inset color bar in all panels beside x axis is thermograph of *P* value of one-way ANOVA used to compare in normalized firing rate (z-score) among the three TSI conditions showed in trends over time (Red: *P* ≤ 0.05; Blue: *P* > 0.05, time interval: 500 ms). The grey transparent shadow in all panels indicates S.E.M. of the y value.


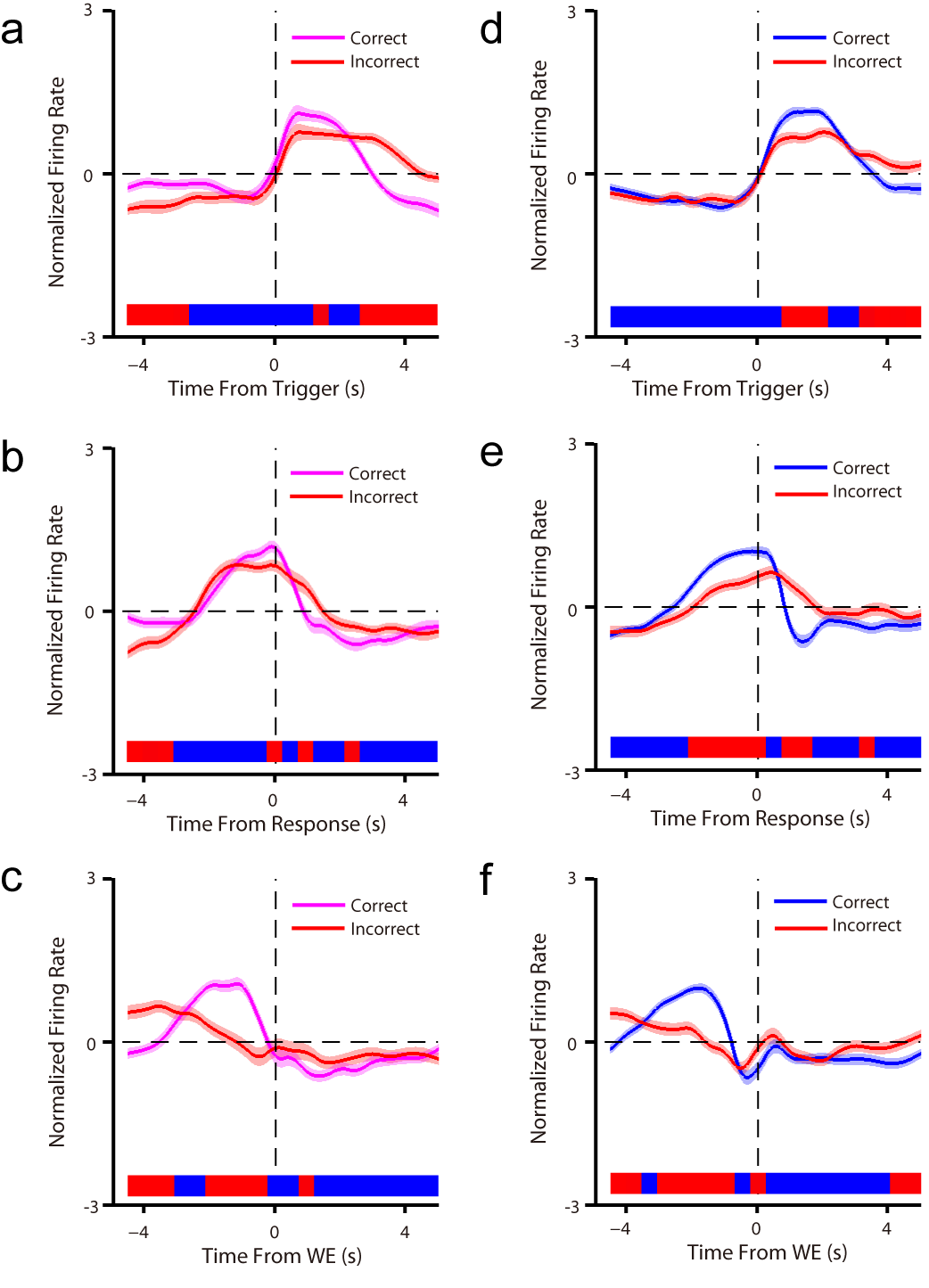


**Figure S9. Comparisons in attention-related neuronal activities between correct and incorrect trials. (a-f)** Averaged trend of normalized firing rate of attention-related activated neurons in correct and incorrect trials (bin = 500ms). (a-c) Visual attention. (d-f) Olfactory attention. **(a, d)** Aligned to the time from trigger. **(b, e)** Aligned to the time from response. **(c, f)** Aligned to the time from WE (the first water port entrance after response). The inset color bar in all panels beside x axis is thermograph of *P* value of one-way ANOVA used to compare in normalized firing rate (z-score) between correct and incorrect trials showed in trends over time (Red: *P* ≤ 0.05; Blue: *P* > 0.05, time interval: 500 ms). The grey transparent shadow in all panels indicates S.E.M. of the y value.


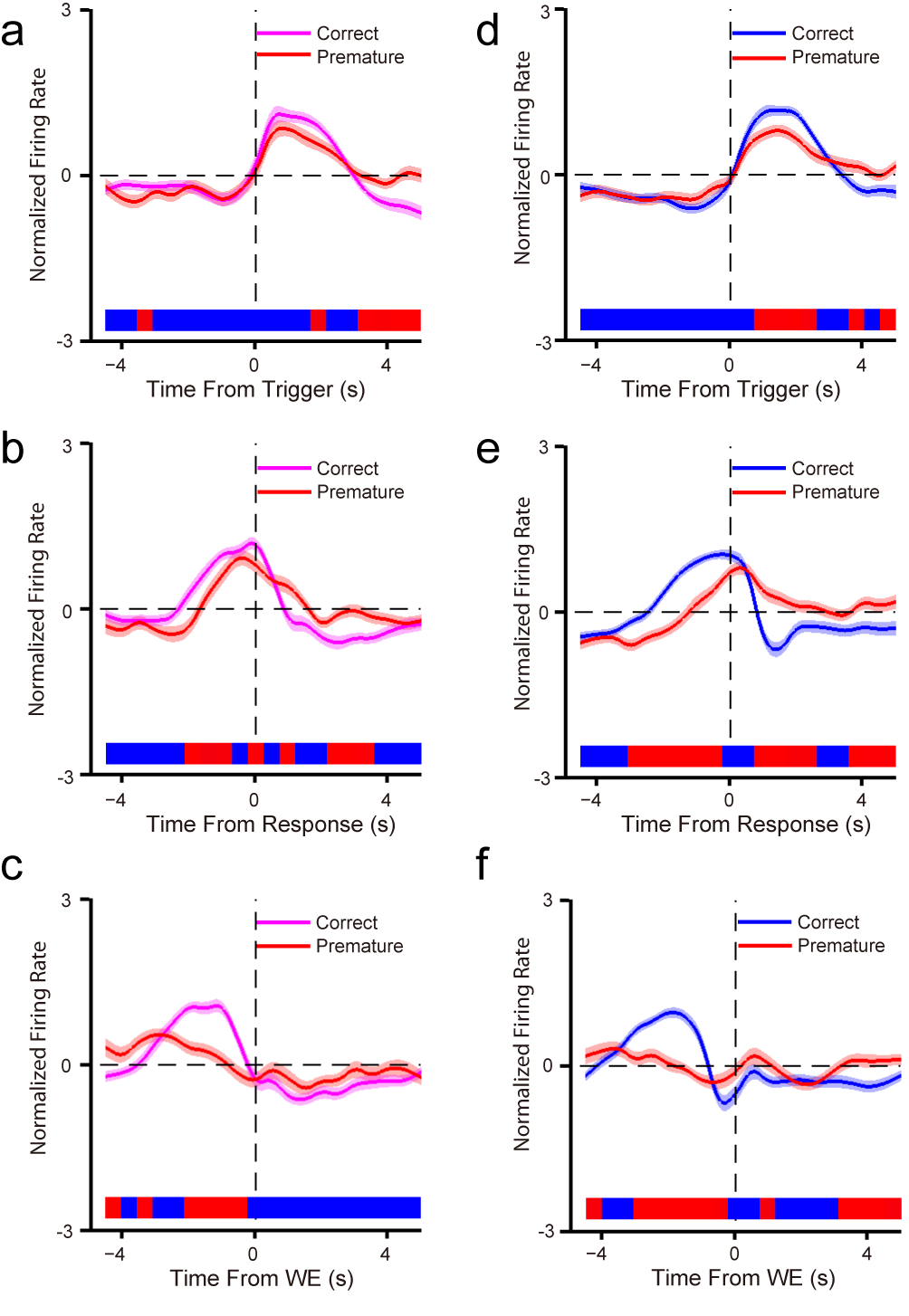


**Figure S10. Comparisons in correlated neuronal activities between correct and premature trials. (a-f)** Averaged trend of normalized firing rate of attention-related activated neurons in correct and premature trials (bin = 500ms). (a-c) Visual task. (d-f) Olfactory task. **(a, d)** Aligned to the time from trigger. **(b, e)** Aligned to the time from response. **(c, f)** Aligned to the time from WE (the first water port entrance after response). The inset color bar in all panels beside x axis is thermograph of *P* value of one-way ANOVA used to compare in normalized firing rate (z-score) between correct and trials with premature responses showed in trends over time (Red: *P* ≤ 0.05; Blue: *P* > 0.05, time interval: 500 ms). The grey transparent shadow in all panels indicates S.E.M. of the y value.


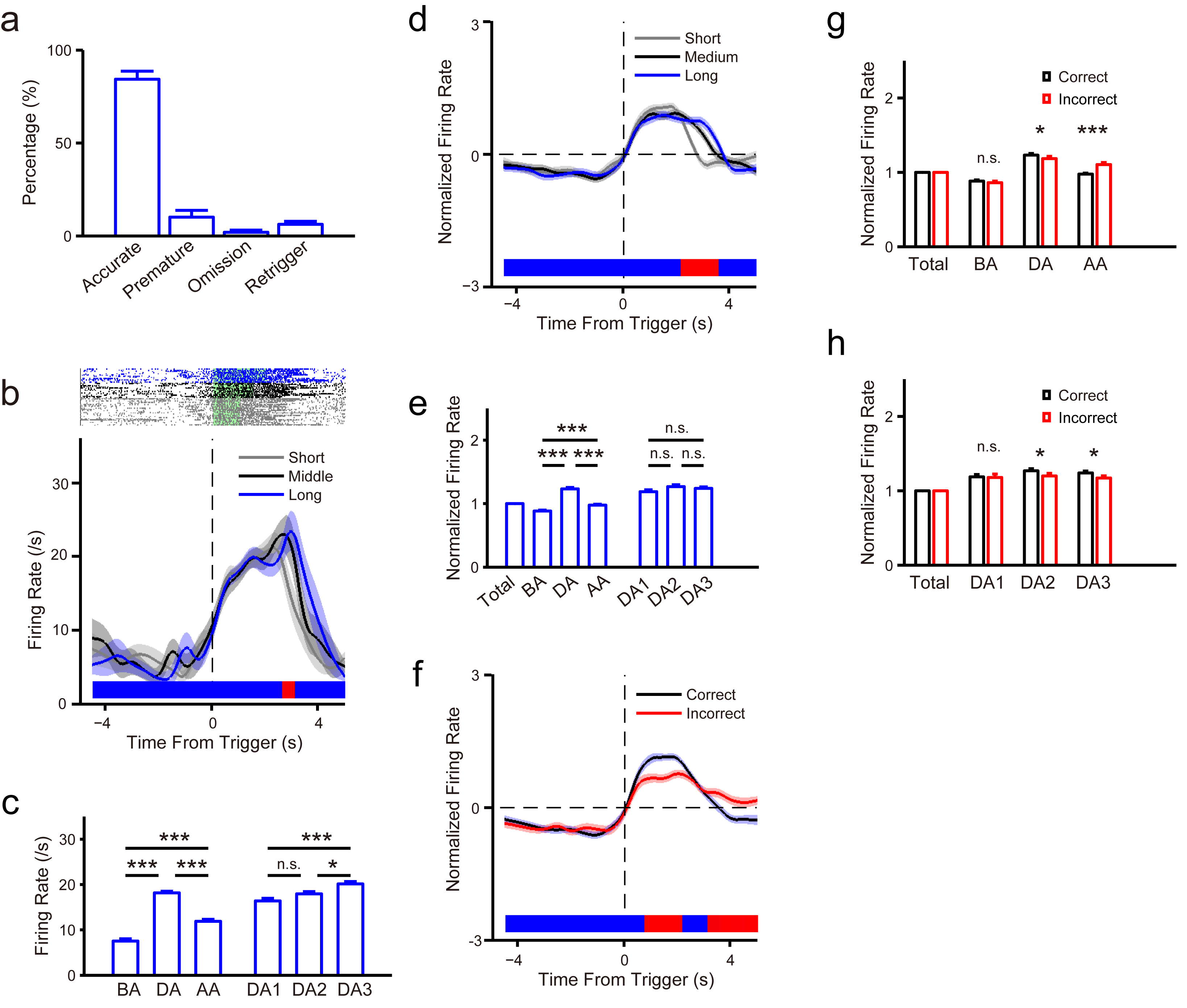


**Figure S11. ACC neuronal activity correlates with olfactory sustained attention.** (a) Behavioral performance in the recording sessions (rat no. = 5). (b) Activities during sustained attention of an attention-related activated neuron (#58). Top: a raster plot of spikes at the 3 TSI values; grey transparent shadow indicates TSI time window; bottom: trend of firing rate in correct trials at 3 TSI values aligned to the time from trigger. (c) Comparisons in firing rate of neuron #58 among three time windows: duration of attention (DA), an interval equaling DA before attention (BA), and the same interval after attention (AA) (one-way ANOVA: *F*(2,216) = 147.86, *P =* 3.51e-41). The DA period is divided into 3 equal, consecutive time windows (DA1, DA2 and DA3), and firing rate among the 3 time windows were compared (one-way ANOVA: *F*(2,216) = 12.42, *P =* 7.84e-6). (d) Population activities during sustained attention of attention-related activated neurons shown by the averaged trend of normalized firing rate in correct trials at 3 TSI values aligned to the time from trigger (n = 66, proportion: 16.06%, all recorded neurons: n = 411). (e) Comparisons in normalized firing rate of all attention-related activated neurons among time windows of BA, DA and AA (one-way ANOVA: *F*(2,195) = 109.05, *P =* 1.63e-32), and among time windows of DA1, DA2, and DA3 (one-way ANOVA: *F*(2,195) = 2.02, *P =* 0.135) (for definition of time windows see c). For each neuron, the firing rate was normalized with mean firing rate of the total time window (from -5 to 5 second aligned to trigger). (f-h) Comparisons between correct and incorrect trials for all attention-related activated neurons (same as d). (f) Trend of normalized firing rate aligned to the time from trigger. (g) Comparisons in normalized firing rate (see e) among the three time windows BA, DA and AA (see c) between correct and incorrect trials (two-way ANOVA with Greenhouse-Geisser adjustment: correctness: *F*(1,65) = 6.09, *P =* 0.016; time: *F*(2,130) = 59.67, *P =* 1.41e-17; correctness * time: *F*(2,130) = 14.86, *P =* 5.45e-6). (h) Comparisons in normalized firing rate among the three time windows DA1, DA2 and DA3 (see c) between correct and incorrect trials (two-way ANOVA with Greenhouse-Geisser adjustment: correctness: *F*(1,65) = 4.47, *P =* 0.038; time: *F*(2,130) = 1.25, *P =* 0.288; correctness * time: *F*(2,130) = 2.90, *P =* 0.062). The inset color bar in panel b, d, and f beside x axis is thermograph of *P* value of one-way ANOVA used to compare in y value among the conditions showed in trends over time (Red: *P* ≤ 0.05; Blue: *P* > 0.05, time interval: 500 ms). The results of multiple comparisons showed in panel c and e were from post hoc of one-way ANOVA with Bonferroni adjustment. The statistic results showed in panel g and h were from post hoc simple effect analyses (MANOVA). The grey transparent shadow in panel b, d and f indicates S.E.M. of the y value. All the statistical data that we showed were mean ± S.E.M. *** *P ≤* 0.001, ** *P ≤* 0.01, * *P ≤* 0.05. n.s. = not significant.


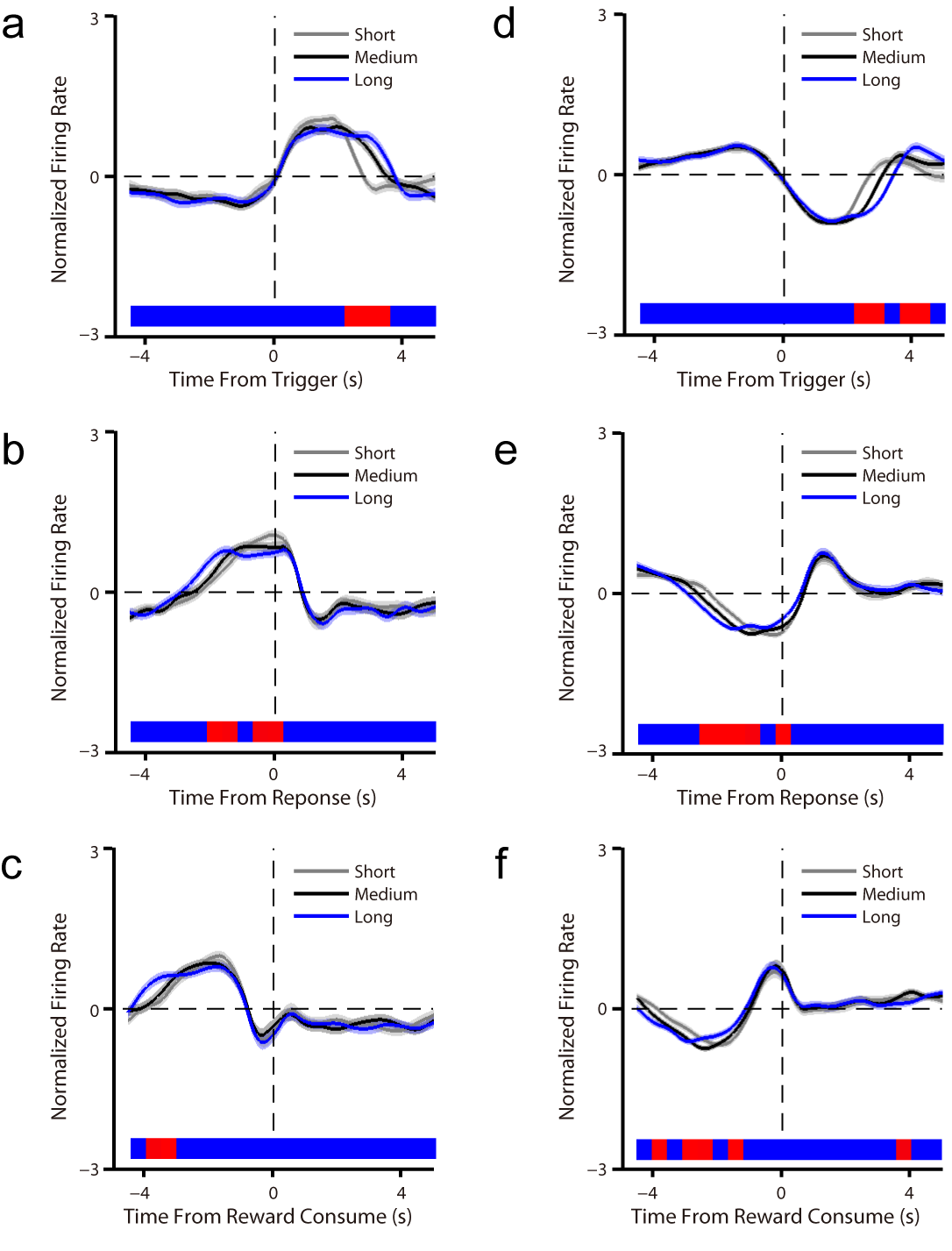


**Figure S12. Activities of two types of neurons during sustained olfactory attention.**

**(a-f)** Averaged trend of normalized firing rate of attention-related neurons in correct trials in the three TSI conditions (bin = 500ms). **(a-c) Activated neurons. (d-f) Inhibited neurons. (a, d)** Aligned to the time from trigger. **(b, e)** Aligned to the time from response. **(c, f)** Aligned to the time from reward consume. The inset color bar in all panels beside x axis is thermograph of *P* value of one-way ANOVA used to compare in normalized firing rate (z-score) among the three TSI conditions showed in trends over time (Red: *P* ≤ 0.05; Blue: *P* > 0.05, time interval: 500 ms). The grey transparent shadow in all panels indicates S.E.M. of the y value.


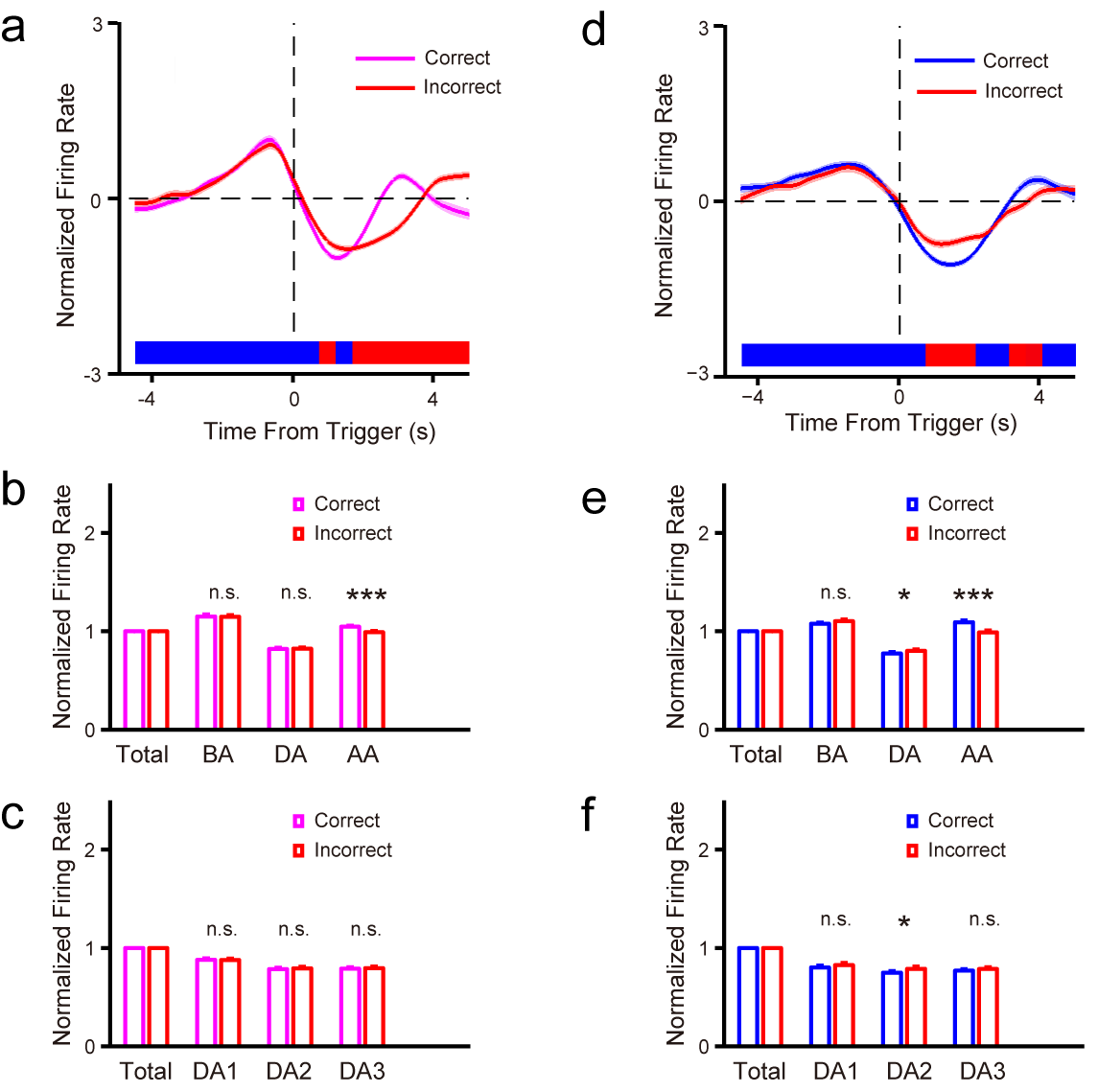


**Figure S13. Comparisons between correct and incorrect trials in firing rate of inhibited neurons in both modalities.**

**(a-c) Visual attention. (d-f) Olfactory attention. (a, d)** Trend of normalized firing rate aligned to the time from trigger. (b-c, e-f) For each time window, the firing rate was normalized with mean firing rate of the total time window (from -5 to 5 second aligned to trigger). (b) Comparisons in normalized firing rate among the three time windows BA, DA and AA (for definition see **Figure S10c**) in visual attention (two-way ANOVA with Greenhouse-Geisser adjustment: correctness: *F*(1,157) = 11.59, *P =* 0.001; time: *F*(2,314) = 121.13, *P =* 2.19e-29; correctness * time: *F*(2,314) = 7.06, *P =* 0.002). (c) Comparisons in normalized firing rate among the three time windows DA1, DA2 and DA3 in visual attention (for definition see **Figure S10c**) (two-way ANOVA with Greenhouse-Geisser adjustment: correctness: *F*(1,157) = 0.23, *P =* 0.633; time: *F*(2,314) = 35.98, *P =* 5.18e-12; correctness * time: *F*(2,314) = 0.22, *P =* 0.799). (e) Comparisons in normalized firing rate among the three time windows BA, DA and AA in olfactory attention (two-way ANOVA with Greenhouse-Geisser adjustment: correctness: *F*(1,127) = 5.26, *P =* 0.023; time: *F*(2,254) = 107.10, *P =* 3.04e-34; correctness * time: *F*(2,254) = 11.52, *P =* 2.49e-4). (f) Comparisons in normalized firing rate among the three time windows DA1, DA2 and DA3 (two-way ANOVA with Greenhouse-Geisser adjustment: correctness: *F*(1,127) = 6.51, *P =* 0.012; time: *F*(2,254) = 4.02, *P =* 0.023; correctness * time: *F*(2,254) = 0.35, *P =* 0.692). The inset color bar in panel a and d beside x axis is thermograph of *P* value of one-way ANOVA used to compare in normalized firing rate (z-score) between correct and incorrect trials over time (Red: *P* ≤ 0.05; Blue: *P* > 0.05, time interval: 500 ms). The statistic results showed in panel b, c, e and f were from post hoc simple effect analyses (MANOVA). The grey transparent shadow in panel a and d indicates S.E.M. of the y value. All the statistical data that we showed were mean ± S.E.M. *** *P ≤* 0.001, ** *P ≤* 0.01, * *P ≤* 0.05. n.s. = not significant.


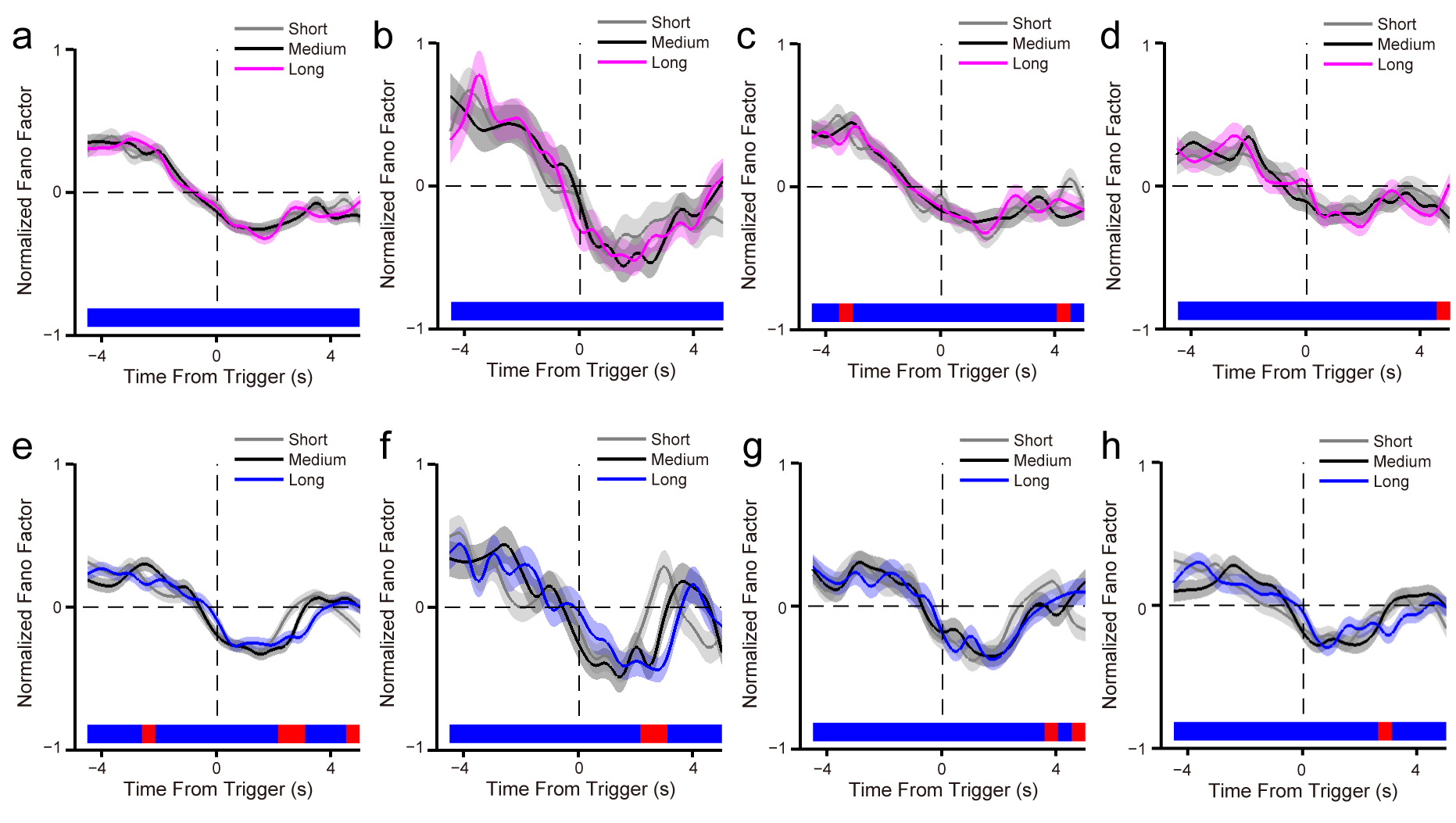


**Figure S14. Trend of normalized fano factor in correct trials for different types of neurons in visual and olfactory attention task.** (a-d) Vision attention. (e-h) Olfactory attention. (a, e) All neurons. (b, f) Activated neurons during attention. (c, g) Suppressed neurons during attention. (d, h) Neurons whose activities did not show any significant changes with attention. The inset color bar in all panels beside x axis is thermograph of *P* value of one-way ANOVA used to compare in normalized fano factor (z-score) among the three TSI conditions over time (Red: *P* ≤ 0.05; Blue: *P* > 0.05, time interval: 500 ms). The grey transparent shadow in all panels indicates S.E.M. of the y value.


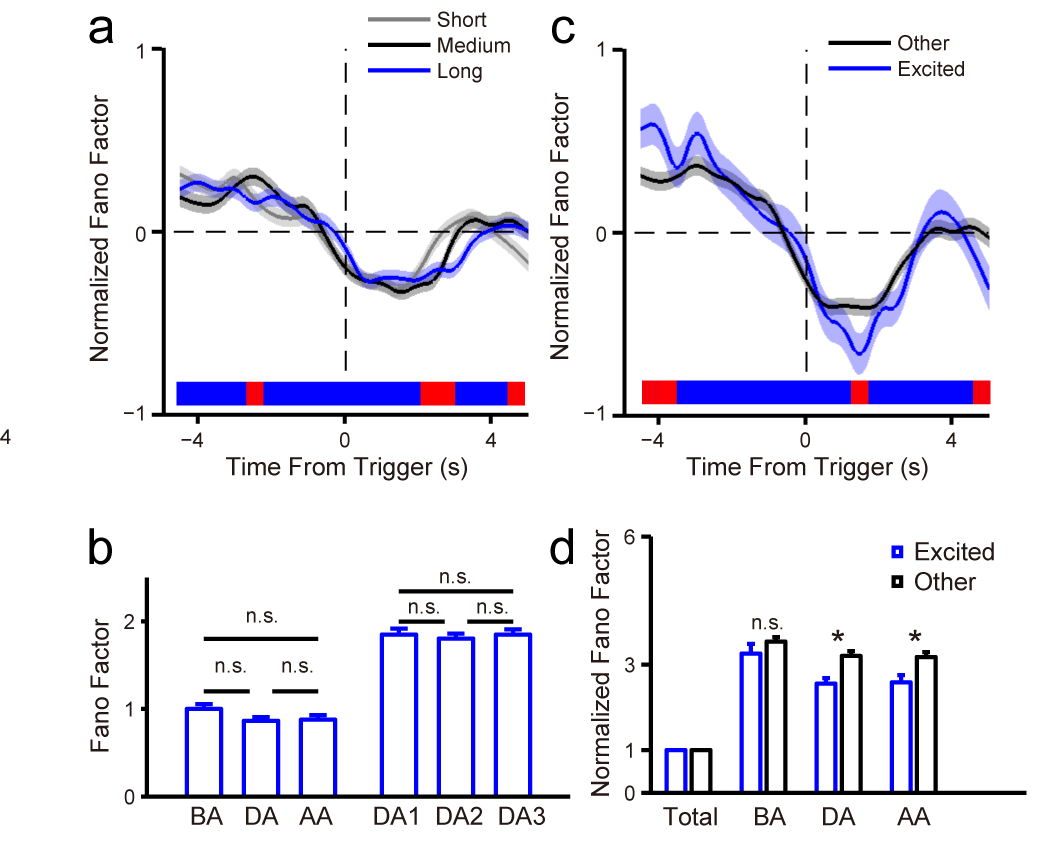


**Figure S15. Fano factor of the recorded neurons in olfactory task was correlated with sustained attention.** (a) Trend of normalized fano factor in correct trials of all recorded neurons at the three TSI values aligned to the time from trigger. (b) Comparisons in fano factor among different time windows (see **Fig. 3** for definitions of time windows) (one-way ANOVA: BA, DA, and AA: *F*(2,1230) = 2.22, *P =* 0.109; DA1, DA2, and DA3: *F*(2,1230) = 0.18, *P =* 0.837). Note that there was no significant difference between time windows of BA and DA when using Bonferroni adjustment in post hoc test, while using LSD as adjustment method, the post hoc test showed marginally significant difference (*P* = 0.055). (c) Trend of normalized fano factor in correct trials for attention-related excited and other neurons aligned to the time from trigger. (d) Comparisons in normalized fano factor (normalized to fano factor for the total time window) in time windows of BA, DA, or AA between excited and other neurons (two-way mixed ANOVA with Greenhouse-Geisser adjustment: time: *F*(2,818) = 19.23, *P =* 8.04e-9; time * neuron type: *F*(2,818) = 1.80, *P =* 0.166). The inset color bar in panel a and c beside x axis is thermograph of *P* value of one-way ANOVA used to compare in normalized fano factor among the conditions showed in trends over time (Red: *P* ≤ 0.05; Blue: *P* > 0.05, time interval: 500 ms). The results of multiple comparisons showed in panel b were from post hoc of one-way ANOVA with Bonferroni adjustment. The statistic results showed in panel d were from post hoc simple effect analyses (MANOVA). The grey transparent shadow in panel a and c indicates S.E.M. of the y value. All the statistical data that we showed were mean ± S.E.M. *** *P ≤* 0.001, ** *P ≤* 0.01, * *P ≤* 0.05. n.s. = not significant.

## SUPPLEMENTARY VIDEOS

**Video S1. Examples of correct trials in the visual test task.**

**Video S2. Examples of correct trials in the olfactory test task.**
